# Type 1 diabetes in 2017: global estimates of incident and prevalent cases in children and adults

**DOI:** 10.1007/s00125-021-05571-8

**Published:** 2021-10-02

**Authors:** Anders Green, Simone M. Hede, Christopher C. Patterson, Sarah H. Wild, Giuseppina Imperatore, Gojka Roglic, David Beran

**Affiliations:** 1 Institute of Applied Economics and Health Research, Copenhagen, Denmark; 2 Steno Diabetes Center Odense, Department of Clinical Research, Odense University Hospital and University of Southern Denmark, Odense, Denmark; 3 Centre for Public Health, Queen’s University Belfast, Belfast, UK; 4 Usher Institute, University of Edinburgh, Edinburgh, UK; 5 Division of Diabetes Translation, Centers for Disease Control and Prevention, National Center for Chronic Disease Prevention and Health Promotion, Atlanta, GA USA; 6 Department of Noncommunicable Diseases, World Health Organization, Geneva, Switzerland; 7 Division of Tropical and Humanitarian Medicine, University of Geneva and Geneva University Hospitals, Geneva, Switzerland

**Keywords:** Adults, Children, Epidemiology, Global estimates, Incidence, Prevalence, Type 1 diabetes

## Abstract

**Aims/hypothesis:**

Data on type 1 diabetes incidence and prevalence are limited, particularly for adults. This study aims to estimate global numbers of incident and prevalent cases of type 1 diabetes in 2017 for all age groups, by country and areas defined by income and region.

**Methods:**

Incidence rates of type 1 diabetes in children (available from 94 countries) from the IDF Atlas were used and extrapolated to countries without data. Age-specific incidence rates in adults (only known across full age range for fewer than ten countries) were obtained by applying scaling ratios for each adult age group relative to the incidence rate in children. Age-specific incidence rates were applied to population estimates to obtain incident case numbers. Duration of diabetes was estimated from available data and adjusted using differences in childhood mortality rate between countries from United Nations demographic data. Prevalent case numbers were derived by modelling the relationship between prevalence, incidence and disease duration. Sensitivity analyses were performed to quantify the impact of alternative assumptions and model inputs.

**Results:**

Global numbers of incident and prevalent cases of type 1 diabetes were estimated to be 234,710 and 9,004,610, respectively, in 2017. High-income countries, with 17% of the global population, accounted for 49% of global incident cases and 52% of prevalent cases. Asia, which has the largest proportion of the world’s population (60%), had the largest number of incident (32%) and prevalent (31%) cases of type 1 diabetes. Globally, 6%, 35%, 43% and 16% of prevalent cases were in the age groups 0–14, 15–39, 40–64 and 65+ years, respectively. Based on sensitivity analyses, the estimates could deviate by ±15%.

**Conclusions**/**interpretation:**

Globally, type 1 diabetes represents about 2% of the estimated total cases of diabetes, ranging from less than 1% in certain Pacific countries to more than 15% in Northern European populations in 2017. This study provides information for the development of healthcare and policy approaches to manage type 1 diabetes. The estimates need further validation due to limitations and assumptions related to data availability and estimation methods.

**Graphical abstract:**

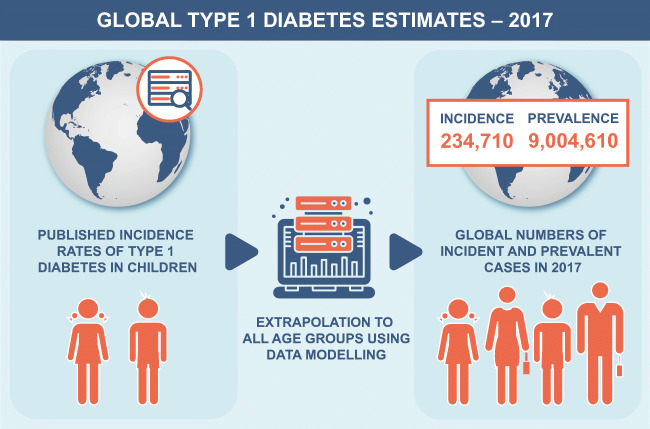

**Supplementary Information:**

The online version contains peer-reviewed but unedited supplementary material available at 10.1007/s00125-021-05571-8.

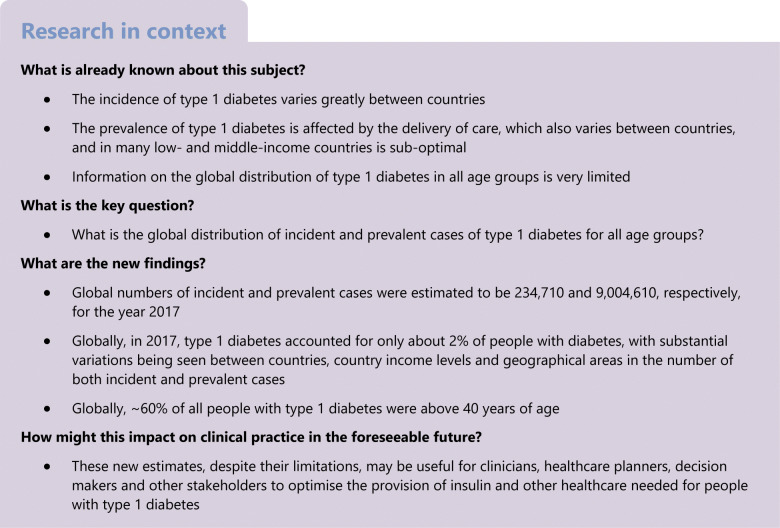



## Introduction

Diabetes is one of the four non-communicable diseases (NCDs) prioritised by the WHO [1]. Although diabetes encompasses both type 1 and type 2 diabetes, type 2 diabetes is given more attention due to its shared risk factors with other NCDs and its larger burden. The WHO estimated that there were 422 million adults with diabetes in the year 2014, without distinguishing between types of diabetes [2]. Type 1 diabetes is defined as absolute insulin deficiency of unknown cause [2]. It has been estimated that type 1 diabetes represents approximately 5–10% of the total prevalence of diabetes, corresponding to 21–42 million people [3, 4]. However, such claims are usually based on northern European populations, which have the highest incidence and prevalence rates of type 1 diabetes. Published data on type 1 diabetes incidence and prevalence in adults are scarce because most of the available literature is restricted to estimates for children and adolescents [5–8]. Data available on the incidence of type 1 diabetes are from incidence registries similar to those established for the WHO Diabetes Mondiale (DIAMOND) study and the Europe and Diabetes (EURODIAB) study, covering children 0–14 years of age and using a standard case definition [9, 10].

The incidence and prevalence of type 1 diabetes differ substantially globally [9, 11] and incidence has been changing over time in different countries [12, 13]. Type 1 diabetes is among the most common chronic diseases in children [14]. Large population-based cohort studies in Europe suggested that life expectancy for people with type 1 diabetes compared with that of the general population was reduced by approximately 13 years for women and 11 years for men at 20 years of age in Scotland and by 18 years for women and 14 years for men among people with type 1 diabetes diagnosed under 10 years of age in Sweden [15, 16]. However, in low-income countries (LICs) and lower middle-income countries (LMICs), high mortality rates are still observed due to lack of access to insulin and adequate healthcare [17, 18].

Without clear data on the overall burden of type 1 diabetes, the planning and establishment of appropriate healthcare are difficult [17, 19]. The aim of this study is to provide country, regional and global estimates of numbers of new cases of type 1 diabetes (incident cases) and numbers of people living with type 1 diabetes (prevalent cases) for 2017, covering all age groups.

## Methods

**Fig. 1:**
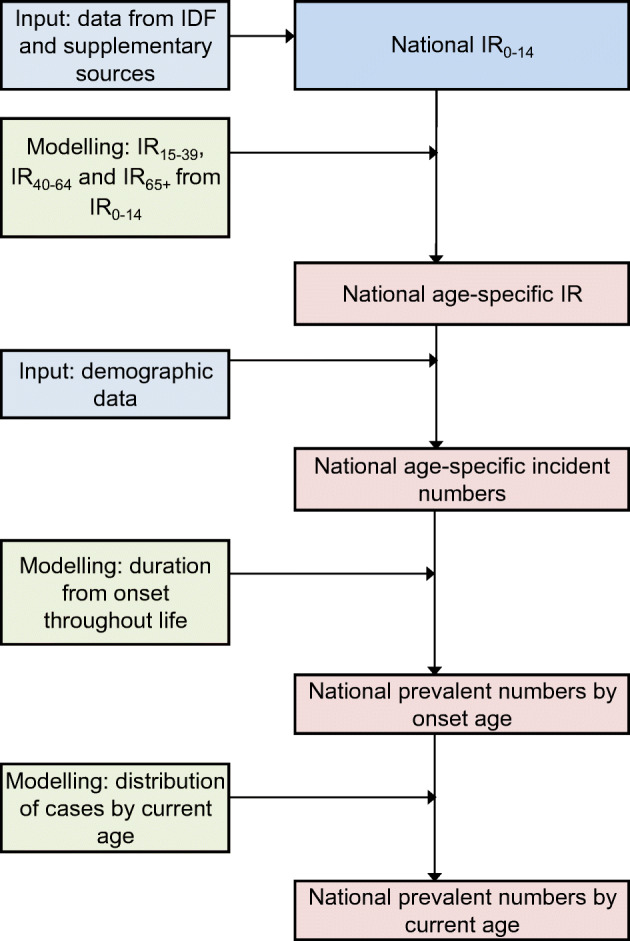
Overview of the approach for estimating numbers of incident and prevalent cases of type 1 diabetes in the world for 2017. IR, incidence rate; IR_0–14_, incidence rate in the 0–14 years age group; IR_15–39_, incidence rate in the 15–39 years age group; IR_40–64_, incidence rate in the 40–64 years age group; IR_65+_, incidence rate in the 65+ years age group

All calculations were done using Microsoft Excel (2016). Figure 1 summarises the estimation process and the electronic supplementary material (ESM) Methods provides a detailed account of the principles and assumptions.

### Overview of the estimation process

Incidence rates for type 1 diabetes in children were derived from peer-reviewed published studies [20]. Most of them used a standardised case definition, defined population and assessment of ascertainment as proposed by the WHO [21] and the EURODIAB study [20]. All incidence rates for type 1 diabetes in childhood (age 0–14 years) used for the present analysis are shown in ESM Table 1. Information on age-specific incidence rates among adults are mainly restricted to high-income countries (HICs) [22, 23], and suggests that the incidence rate among adults is considerably lower than that among children. For this analysis, we used Danish data for the year 2017, established as an update of a previously published dataset [24], as further explained in ESM Table 2, ESM Table 3 and ESM Fig. 1.

Demographic data on age group and country-specific population estimates for the year 2017 were obtained from the United Nations (UN) [25]. Countries were grouped according to level of income as classified by the World Bank [26], as well as by major geographical area and sub-area/region as used in UN World demography [25]. Due to paucity of sex-specific data on incidence, sex was not included in the estimation model.

Throughout the analysis age was grouped into four categories (0–14, 15–39, 40–64 and 65+ years), as a compromise between the desire to provide estimates for narrowly defined age groups vs the lack of sufficiently detailed epidemiological data on type 1 diabetes. This grouping was chosen since it covers childhood (0–14 years, which is covered best by the epidemiological information available), younger adulthood (15–39 years), middle age (40–64 years) and older age (65+ years).

### Estimating incident numbers of type 1 diabetes

Published incidence data for type 1 diabetes were identified for 90 countries [20]. There were only three studies from 30 LICs. If no study was available for a particular country, the country was assigned an incidence rate from another country, based on geographical proximity, per capita income, study quality and ethnic background [20]. Concerning incidence rates for ages beyond childhood and young adulthood, a global review [6] found that among 70 identified publications on the epidemiology of type 1 diabetes, 32 studies covered age groups above 30 years, but only six covered age groups above 60 years [6]. In general, the incidence rate for the age group 15–39 years was about half that for children aged 0–14 years, and age groups above 40 years were lower. In a study covering 10% of the Chinese population [27], the incidence rates of type 1 diabetes for the age groups 15–39, 40–64 and ≥65 years were 0.57, 0.28 and 0.19 times the incidence rate seen in children aged 0–14 years. To provide estimates of incidence rates in adults, country-specific incidence rates of type 1 diabetes in children aged 0–14 years were used together with assumed ratios of incidence rates in older age groups compared with incidence rates in children aged 0–14 years. These scaled age-specific rates were applied to population estimates to provide age-specific incident case numbers for each country. Specifically, the following age-specific incidence rate ratios were obtained from Danish data (see ESM Table 2) and applied globally: age group 0–14 years, 1.00 (used as reference); age group 15–39 years, 0.50; age group 40–64 years, 0.30; age group 65+ years: 0.25. The age-specific incidence rates were then applied to demographic data to estimate incident case numbers stratified by onset age.

### Estimating duration of type 1 diabetes

This requires estimates of mean remaining lifetime from onset age of type 1 diabetes. Since this is likely to depend on the health and socioeconomic conditions of a country, a penalty function, based on the country’s under 5-years child mortality rate (CM), was generated and applied to adjust life expectancy in people with type 1 diabetes relative to that from Danish data (see ESM Fig. 1). CM values were chosen as an alternative to life expectancy functions covering the full lifespan, as CM is largely independent of the impact of mortality attributable to HIV/AIDS. Country-specific values of CM (per 1000 live births) for the year 2017 were obtained from the UN Inter-agency Group for Child Mortality Estimation [28] and are listed in ESM Table 1.

The penalty (Pen) for a given country was calculated as the CM (per 1000) divided by the constant of 130, selected as just above the highest CM for any country in 2017 [28], as follows:

$$ \mathrm{Pen}=\mathrm{CM}/130 $$

The penalty value (listed by country in ESM Table 1) was used to scale the mean duration of type 1 diabetes within an age band for a given country relative to what is considered the maximum mean duration under optimal conditions of living with type 1 diabetes. This was accomplished using the relationship:

$$ \mathrm{Mean}\ \mathrm{duration}=\mathrm{global}\ \mathrm{maximum}\ \mathrm{mean}\ \mathrm{duration}\times \left(1\hbox{--} \mathrm{Pen}\right) $$

Global maximum mean duration was taken as 60.4, 43.4, 21.1 and 8.5 years based on estimated remaining lifetimes for Danish individuals with assumed type 1 diabetes with onset in age groups 0–14, 15–39, 40–64 and 65+ years, respectively. These estimations are in close agreement with data from Scotland [15] (see ESM Table 2 and ESM Table 3 for details). A global minimum mean duration was set at 0.5 years reflecting assumed short survival (regardless of age) in people diagnosed with type 1 diabetes in countries where people have severe challenges accessing insulin or facilities capable of diagnosing and managing type 1 diabetes.

### Estimating prevalent case numbers

To estimate prevalence from incidence, we used the general principle:

$$ \mathrm{Prevalence}=\mathrm{incidence}\times \mathrm{mean}\ \mathrm{duration} $$

The equation assumes epidemiological ‘steady state’ (i.e. that the annual number of new cases equals the annual number of deaths from the patient population). The principle was applied for a given country to estimate the prevalence of type 1 diabetes according to age at onset and to provide estimates of the prevalence of type 1 diabetes according to attained age. Using the steps outlined above, country-specific numbers were obtained for prevalence by onset age (see ESM Table 4 for an example). To obtain numbers of prevalent cases by attained age a further step is necessary. This was accomplished by allocating the remaining years of life to age classes using cumulative survival from onset of type 1 diabetes to the end of each of the subsequent age intervals considered, assuming epidemiological ‘steady state’ and with penalty adjustment as before. The aforementioned Danish data provided estimated mean age at onset within each of the age intervals 0–14, 15–39, 40–64 and 65+ years, and these were applied globally. The same data also provide estimated cumulative survival from age at onset to the end of each of the subsequent age intervals for people with age at onset in the intervals 0–14, 15–39 and 40–64 years (ESM Table 5). For each group defined by attained age, the prevalent number was obtained from cumulating contributions from people according to their onset age. Example estimation data are shown in ESM Table 6 and ESM Fig. 2. Prevalence estimates were age-standardised according to the WHO world standard population for 2000–2025 [29].

### Sensitivity analyses

Four sensitivity analyses were performed to investigate the effects of changing assumptions concerning the penalty function, the assumed maximal life expectancy for each age group at onset, as well as two different scenarios for the ratios of age-specific incidence rates relative to the rate in childhood. The scenarios covered by the sensitivity analyses are specified in ESM Table 7.

### Consultation with WHO member states

Following initial estimates using this approach, a 12 week formal consultation with all WHO member states in 2019 was initiated. All countries were invited to comment on the first draft of estimates and share additional data or provide additional information about national data sources.

## Results

Thirty-seven countries provided feedback on the original estimates. Although several countries maintain national, regional or local diabetes registries, there were few published reports and the definitions of type 1 diabetes varied. In many instances, type 1 diabetes was identified solely on the basis of current insulin treatment. One large country provided new data that were eligible for inclusion, reducing the initial global prevalence estimate of the number of people with type 1 diabetes by 3046.

**Fig. 2:**
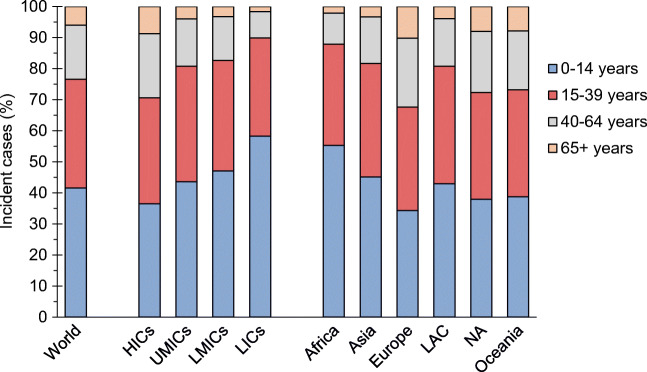
Distribution of incident cases of type 1 diabetes by age at onset and by income groups and regions defined by UN population estimates. LAC, Latin America and the Caribbean; NA, North America

The number of people who developed type 1 diabetes in 2017 (incident cases) was estimated to be 234,710 (Table 1 and ESM Table 8). HICs accounted for 115,600 cases (49% of the global incidence but only 17% of the population). Asia, which has 60% of the world’s population, was the continent with the largest number of incident cases (74,390, 32%), followed by Europe (62,360, 27%) with 10% of the world’s population. Figure 2 shows the age distribution of the incident cases by income group and geographical area. Globally, 97,580 cases (42% of all incident cases) were estimated to occur below 15 years of age, ranging from 34% for Europe to 55% in Africa. Worldwide 14,080 incident cases (6% of the total) were estimated to occur in people over 64 years of age, the proportions ranging from 2% in Africa to 10% in Europe.

**Table 1:** Global estimates of numbers of incident and prevalent cases of type 1 diabetes for 2017 and prevalence proportions by income level and regions used in UN population estimates, grouped by age in years

Variable	Incident cases (in 1000s)					Prevalent cases (in 1000s)					Prevalence (per 1000)	
	0–14 years old	15–39 years old	40–64 years old	65+ years old	Total	0–14 years old	15–39 years old	40–64 years old	65+ years old	Total	Crude	Standardised^a^
World	97.58	82.23	40.83	14.08	234.71	541.18	3107.77	3905.22	1450.44	9004.61	1.20	1.19
Income level												
HICs	42.15	39.49	23.91	10.05	115.60	247.59	1530.47	2026.34	843.60	4648.01	3.59	3.27
UMICs	18.40	15.70	6.42	1.69	42.21	103.54	601.69	739.86	261.99	1707.07	1.78	1.77
LMICs	32.65	24.65	9.86	2.22	69.38	169.73	890.10	1050.45	323.86	2434.15	0.57	0.57
LICs	4.38	2.38	0.64	0.12	7.52	20.32	85.51	88.57	20.98	215.39	0.22	0.28
Region												
Africa	12.80	7.53	2.33	0.48	23.13	62.88	289.72	314.37	88.63	755.59	0.60	0.84
Asia	33.56	27.20	11.16	2.47	74.39	179.89	990.31	1195.99	387.31	2753.51	0.62	0.60
Europe	21.39	20.79	13.87	6.31	62.36	126.05	790.94	1071.27	461.47	2449.73	3.30	3.00
LAC	12.16	10.71	4.32	1.11	28.30	68.87	406.01	501.50	178.16	1154.53	1.80	1.79
NA	16.36	14.84	8.51	3.44	43.15	95.73	583.52	760.88	309.77	1749.89	4.85	4.44
Oceania	1.31	1.17	0.64	0.26	3.39	7.76	47.28	61.21	25.12	141.36	3.55	3.37

^a^Standardised to the WHO world standard population 2000–2025

LAC, Latin America and the Caribbean; NA, North America

**Fig. 3:**
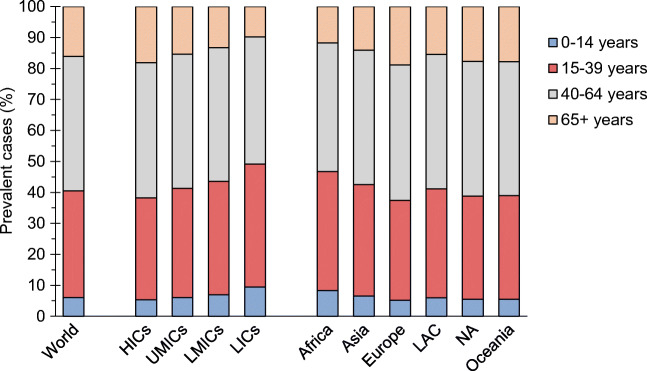
Distribution of prevalent cases of type 1 diabetes by current age and by income groups and regions defined by UN population estimates. LAC, Latin America and the Caribbean; NA, North America

There were an estimated 9,004,610 prevalent cases of type 1 diabetes in 2017 globally (Table 1 and ESM Table 8). Six per cent of prevalent cases were in the 0–14 years age group, 35% in the 15–39 years age group, 43% in the 40–64 years age group and 16% in the 65+ years age group (Fig. 3). HICs accounted for 52% of total prevalent cases, with upper middle-income countries (UMICs), LMICs and LICs accounting for 19%, 27% and 2% of prevalent cases, respectively. Of the total number of estimated prevalent cases of type 1 diabetes, 31% were in Asia, followed by 27% in Europe, 19% in North America, 13% in Latin America and the Caribbean, 8% in Africa and 2% in Oceania. Age-standardised prevalence was highest in North America (4.4 per 1000), followed by Oceania, which is dominated by Australia and New Zealand (3.4 per 1000), and Europe (3.0 per 1000), and lowest in Africa (0.8 per 1000) and Asia (0.6 per 1000).

### Sensitivity analyses

ESM Table 7 specifies the details of the four sensitivity analyses performed separately; in one of the analyses, the penalty function was not applied (‘ignoring penalty function’), in another, life expectancy was reduced (‘reduced mean duration’) and followed by two different sets of scaling values of incidence rates from childhood to older age groups (‘changed incidence scaling 1’ and ‘changed incidence scaling 2’).

In the first sensitivity analysis (‘ignoring penalty function’) all parameters were equal to those of the core model with the exception that the penalty function was not applied. In this scenario, which reflects the situation where all people with type 1 diabetes have optimal healthcare, the prevalence estimate would increase globally by 16% but with marked variability, ranging from +3% in Oceania to +52% in Africa, and from +4% in HICs to +77% in LICs (see ESM Table 9). The results in this scenario may be interpreted as the largest number of prevalent cases of type 1 diabetes that could be attained given current incidence levels and penalty similar to countries with the lowest CMs. Incident case estimates remained unaffected in this sensitivity analysis.

In the second sensitivity analysis (‘reduced mean duration’) all estimates of mean life expectancy from onset were reduced while maintaining the penalty function and the child/adult incidence ratios (as in the core model). In this scenario, which illustrates the impact of gross overestimation of mean disease duration in the core model, the global prevalence estimate was reduced by 28%, ranging from −27% to −29% by geography and country income group (ESM Table 9). Incident case estimates remained unaffected in this sensitivity analysis.

In the first of the child/adult incidence ratio sensitivity analyses (‘changed incidence scaling 1’), scaling was altered to reduce incidence rates in older age groups while maintaining all other parameters of the core model. The results are summarised in ESM Table 9. Globally, the number of incident cases was reduced by 20%, ranging from −22% in Europe to −16% in Africa and from −15% in LICs to −21% in HICs. The prevalence was reduced by 16% globally, ranging from −17% in Europe to −13% in Africa and between −12% and −17% by income group.

The second child/adult incidence ratio sensitivity analysis (‘changed incidence scaling 2’) assumed that the incidence rates for all age groups above childhood were scaled at 0.5 relative to the incidence in childhood. Here, incident cases were increased by 18% globally, ranging from +9% in Africa to +25% in Europe and from +7% in LICs to +22% in HICs (ESM Table 9). Prevalent cases were increased by 7% globally, ranging from +4% in Africa to +10% in Europe and from +3% in LICs to +9% in HICs.

## Discussion

Our estimates find substantial variations in the number of both incident and prevalent cases of type 1 diabetes between countries, country income levels and geographical areas. Prevalence is about ten times higher in HICs vs LICs. North America has the highest age-standardised prevalence at 4.4 per 1000 vs Asia with only 0.6 per 1000 (Table 1). These estimates also suggest that, globally, 60% of all people with type 1 diabetes are above 40 years of age.

The WHO has estimated that there were 422 million people aged 18 or more years with diabetes in the year 2014. Adding our estimates of type 1 diabetes in children under 15 years, our global estimate of about 9 million people with type 1 diabetes represents around 2% of the estimated total cases of diabetes [2]. Using IDF estimates of the global diabetes burden of 425 million in people 20–79 years old in 2017 does not markedly change this proportion [30]. Based on the WHO and IDF estimates and our results, the proportion of total burden attributable to type 1 diabetes varies considerably between populations. For example, in China type 1 diabetes represents less than 1% of all prevalent cases of diabetes in contrast to the UK and Finland where it is estimated to account for 8.6% and 17%, respectively. These latter figures are consistent with previous estimates of 8% [31] and 15% [32]. The missing numbers of cases of type 1 diabetes in people aged 15–17 years and those with type 2 diabetes under the age of 18 years are unlikely to be sufficiently large to affect the above estimate of the proportion of type 1 diabetes in total diabetes [33].

Two existing approaches have described the epidemiology of type 1 diabetes. The Global Burden of Disease (GBD) study [34] has published worldwide estimates of incident and prevalent cases for type 1 diabetes. For many countries, the estimates differ substantially from our estimates, with some unusually high estimates of numbers of prevalent cases given the estimated number of incident cases. The GBD study included ‘cases of diabetes that are on insulin’ and could thus include people with type 2 diabetes or gestational diabetes in addition to type 1 diabetes. Estimates from the IDF are only for children and adolescents and, as detailed above, use data from other countries when context-specific data are lacking. Therefore, our study is the first to more rigorously estimate the global burden of type 1 diabetes for all ages using the standard case definition of type 1 diabetes, taking an innovative approach.

Limitations of our approach include the possibility that the underlying assumption of epidemiological equilibrium (Prevalence = Incidence × Duration) may be violated because of a global pattern of increasing incidence rates. This equilibrium may be further disrupted by improving prognosis of type 1 diabetes [35]. Accordingly, the estimates of prevalent numbers of people with type 1 diabetes must be considered as tentative. The quantification of the impact of such sources of error requires detailed data on incidence, prevalence and mortality of type 1 diabetes over a long period of time for representative individual countries, however, such data are currently lacking. This study had to address gaps in knowledge by making multiple assumptions that need to be tested further. The incidence of type 1 diabetes in children in most LICs and LMICs is unknown and there is even greater paucity of data on the incidence rates in different age groups and on the mortality or life expectancy in people with type 1 diabetes. Moreover, only few studies provide national estimates. To address these limitations, extrapolations have been made across countries for incidence estimates and wider extrapolation of age scaling of incidence beyond 14 years of age has been applied using data from Denmark. Twelve studies from which estimates of incidence were derived are more than 20 years old and probably underestimate the current incidence of type 1 diabetes. Furthermore, using CM as a ‘penalty adjustment’ affects the estimates for LICs and LMICs in particular, and may not capture all the differences between countries in survival rates of people with type 1 diabetes. Further relevant analyses, including stratification by sex and urban/rural residence, would require additional incidence and mortality data, which are not currently available. Based on the sensitivity analyses, we believe the core model represents realistic global estimates of the incidence and prevalence of type 1 diabetes for the year 2017 with an uncertainty interval of about ±15%.

Because of the limitations mentioned above, our results need validation. Nevertheless, we believe that our methodological approach may be of both scientific interest and practical use in adding to the global knowledge of the epidemiology of type 1 diabetes. Clear gaps exist in the descriptive epidemiology of type 1 diabetes, including how its causes affect incidence and its survival rate influences prevalence between different regions in the world. LICs have a particularly marked paucity of data. This lack of data has an impact on health system and policy responses, which are important given the deficiencies in healthcare provision for diabetes diagnosis and management.

There are opportunities for many countries to develop health system responses to better address the needs of people with type 1 diabetes across all stages of the life course [36]. Currently, in both high- and low-income settings, the provision of care for type 1 diabetes is mainly centralised in hospitals located in capital cities or large urban areas, and a more decentralised approach could be considered. Some donation programmes have addressed the challenges of managing type 1 diabetes, even in low-income settings, but more needs to be done to ensure sustainable solutions are found [37]. As 2021 marks the centenary of the discovery of insulin, access to insulin remains problematic in many low-income populations, both in less developed and in more developed countries, and is a major contributor to decreased life expectancy [17, 19].

With the global agenda focusing on the prevention of type 2 diabetes and universal health coverage, type 1 diabetes presents national and global policy makers with a conundrum on how to manage a complex chronic condition requiring a medicine essential for survival, as well as a wide range of services to reduce risk of complications that cannot necessarily be provided at a primary healthcare level. In all countries the high and rapidly increasing burden of type 2 diabetes means that the contribution of type 1 diabetes to global diabetes prevalence is lower than previously suggested [3, 38], but the years of life lost to type 1 diabetes are disproportionately higher than suggested by the numbers of cases. Additionally, although much emphasis is placed on paediatric management of type 1 diabetes, this study shows that this population, although important, does not account for the majority of the global burden of type 1 diabetes as 43% of total prevalent cases of type 1 diabetes were found to be in the 40–64 years age group. These findings suggest that global responses targeted at improving type 1 diabetes care should ensure these older populations are included.

Given the global inequity in access to insulin, delivery systems and technologies for the diagnosis, care and management of type 1 diabetes, the global community should consider developing appropriate responses addressing clinical and programmatic interventions to help improve access to insulin and organisation of care. To do this effectively, gaps in knowledge in the epidemiology of type 1 diabetes need to be addressed as highlighted by this study.

## Supplementary Information

ESM 1 (PDF 706 kb)

## Data Availability

All data generated and analysed during this study are included in this published article (and its supplementary information file).

## Abbreviations

CM: Child mortality rate
EURODIAB: Europe and Diabetes
GBD: Global Burden of Disease
HIC: High-income country
LIC: Low-income country
LMIC: Lower middle-income country
NCD: Non-communicable disease
UMIC: Upper middle-income country
UN: United Nations
