# Supplementary material for: Type 1 diabetes in 2017: global estimates of incident and prevalent cases in children and adults
Source: Diabetologia. 2021 Oct 2;64(12):2741–50. doi: 10.1007/s00125-021-05571-8 (PMC8563635; doi:10.1007/s00125-021-05571-8)

Electronic Supplementary Material:
Green A et al: Type 1 diabetes in 2017: global estimates of incident and prevalent cases in children and adults

Methods

This section describes data input and modelling techniques used for producing the global estimates of type 1 diabetes incidence and prevalence.

In this study, type 1 diabetes is defined as absolute insulin deficiency of unknown cause and a consequent need for insulin treatment for survival.

Overview of the estimation process

Information on incidence and prevalence of type 1 diabetes in all age groups is lacking for most countries whereas incidence rates in children have been reported for many countries. To obtain prevalence estimates the first step is to model the incidence rates in older age groups using the rate assumed for children. The second step is to estimate prevalence from incidence under specified assumptions of life expectancy, i.e. the mean duration from onset of type 1 diabetes until certain age levels and, ultimately, death. The mean duration depends not only on the age at onset but also on the conditions for survival in a given country, particularly concerning access to insulin treatment and other types of healthcare needed to manage type 1 diabetes. Therefore, the mean duration for a given age at onset has been adjusted by a penalty function. The penalty function assigns high values to countries with poor circumstances and low values to countries with good circumstances, as described below.

To estimate prevalence from incidence the general principle of

$$\text{Prevalence} = \text{Incidence} \times \text{Mean Duration}$$

has been used. The equation assumes a situation of epidemiological ‘steady state’, i.e. that the annual number of new cases equals the annual number of deaths from the patient population of persons with type 1 diabetes. With increasing incidence rates and improving prognosis in type 1 diabetes this assumption is violated. To our knowledge there are no data available to quantify this bias for various types of countries but because the attained prevalence population includes cohorts of patients diagnosed in periods with lower incidence levels and poorer prognosis the numbers of prevalent cases in our report may represent overestimates.

The principle of estimating prevalence from incidence and duration has been applied in two different contexts for a given country: First, to obtain an estimate of the total prevalence of type 1 diabetes from the summation of prevalence estimates stratified by age at onset. Second, to stratify the total prevalence by attained age.

Estimating incident numbers of type 1 diabetes

The country-specific incidence rates for children aged 0-14 years published recently in the 9th edition of the IDF Diabetes Atlas [1] have been made available for the present analysis. All incidence rates of type 1 diabetes in childhood (age 0-14 years) applied in the present analysis are shown in ESM Table 1 (right column) with countries grouped by continent and major area. Global demographic data, referring to year 2017, have been obtained from the United Nations (UN) Population Division [2].
Grouping of countries has been done according to various classifications, including

- By major geographical area and sub-area/region as used in UN World Population Prospects 2019 [2]
- By category of per-capita income as classified by the World Bank [3]

ESM Table 1 contains the country-specific incidence rates (age group 0-14 years) used, together with reference to the data sources [4 - 66] and the relevant country-specific characteristics.

Age grouping
Throughout the analysis age has been grouped by four categories: 0-14; 15-39; 40-64; 65+ years as a compromise between the desire to provide estimates for narrowly-defined age-groups versus the lack of sufficiently detailed epidemiological data on type 1 diabetes.

Due to paucity of data on sex-specific incidence and mortality rates in type 1 diabetes, the present analysis has been performed without stratification by sex.

Incidence rates of type 1 diabetes in adult age groups
Due to the lack of data on incidence rates of type 1 diabetes beyond childhood we have for this analysis used Danish data for the year 2017, established as an update of a previously published dataset established on the basis of Danish central health registers [67]. In brief, the population of diabetes (regardless of type) has been ascertained from prescription data on antidiabetic drugs and admissions due to diabetes from hospital registrations. Patients with type 1 diabetes have been identified if insulin treatment was commenced within one year after the first registration qualifying for diabetes (the within one year part of the criterion was applied only for patients with encounters relevant to diabetes after 1995 due to restrictions in data availability).
For the years 2016 and 2017, the annual number of incident cases of type 1 diabetes in Denmark was approximately 900 in a population of about 6.5 million. The number of prevalent cases was approximately 26,000 in both 2016 and 2017.
The incidence rates for age groups beyond childhood have been scaled relative to that applied for children (aged 0-14 years). Using the Danish age-specific incidence rates for the years 2016 and 2017 scaling values have been obtained as shown in ESM Table 2 and applied globally. The effects of applying alternative scaling factors are investigated in the sensitivity analyses.

Introducing the penalty function
The estimation model adjusts for the fact that life expectancy in type 1 diabetes (mean duration from onset of type 1 diabetes) depends on the general living conditions for a given country by introducing a ‘penalty’ (Pen). High and low values of Pen indicate poor and good circumstances, respectively. Based on recommendations from World Health Organization, rates of childhood mortality (CM) in children under 5 years old have been used as input to the penalty function. Country-specific values of CM have been obtained from data referring to 2017 as published by the United Nations Inter-Agency Group for Child Mortality Estimation (IGME) [68] and reproduced in ESM Table 1.
The value of Pen for a given country is estimated as

$$Pen_{country} = CM_{country} \text{ (per 1000 livebirths)}/130$$

The constant 130 has been selected because it is just above the highest CM rate reported by UN for 2017. Formulated this way, the Pen-value increases with increasing CM. For example, taking the value of CM for Sierra Leone at 110.5 per 1,000 gives a Pen-value at 0.85. In contrast, for a country like New Zealand, with a CM at 5.3 per 1,000, the Pen-value is 0.041.

Estimating duration of type 1 diabetes

As explained above, the basic principle for the estimation of prevalence is to use age-specific incidence and average duration from diagnosis given age at diagnosis. The Pen-value is applied to the estimation of mean durations in type 1 diabetes by:

$$\text{Mean duration}_{\text{country}} = \text{Maximum mean duration} \times (1 - \text{Pen}_{\text{country}}).$$

The maximum mean duration represents the mean remaining life-years expected for a patient with type 1 diabetes living under optimal circumstances, from disease onset. Mortality experiences from type 1 diabetes in Denmark during the years 2015-2017 have been used according to an example from Scotland [69]. Within each of the age groups 0-14, 15-39, 40-64, and 65+ years, the mean age at onset was obtained from the Danish incident cases registered for the period 2015-2017. The corresponding estimated remaining life-years from the mean onset ages were obtained from the data as shown in ESM Fig.1. Finally, since a Pen-value at 0.033 has been assigned to Denmark (ESM Table 1), the Danish remaining life-years, given mean age at onset within each of the four age groups) were divided by 1-0.033 (=0.967) to obtain the maximum mean durations of type 1 diabetes at 60.4, 43.4, 21.1 and 8.5 years for each of the age groups 0-14, 15-39, 40-64, and 65+ years (ESM Table 3).

A global minimum mean value has been set at 0.5 year reflecting the assumed short survival (regardless of age) in persons diagnosed with type 1 diabetes in countries without access to insulin treatment and other healthcare support for persons with type 1 diabetes.

Estimating prevalent case numbers

Prevalence numbers by age at onset

It is assumed that the country-specific prevalence for patients with a given onset age group is estimated by

$$\text{Prevalence}_{\text{onset age group}} = \text{Incidence}_{\text{onset age group}} \times \text{Duration}_{\text{onset age group}}$$

applied for the age groups 0-14, 15-39, 40-64, and 65+ years. Age-specific incidence rates are those scaled from the age-group 0-14 years for the country concerned (see ESM Table 1) and presented in ESM Table 2. Duration is obtained as the maximum mean duration for each age at onset group (see ESM Table 3), adjusted for the penalty assigned to the country concerned (ESM Table 1). ESM Table 4 shows the steps in estimating prevalence by age at onset using New Zealand as an example country.

Prevalence numbers by attained age

To derive prevalence estimates by attained age from prevalence estimates by age at onset we apply the principle that, assuming a state of epidemiological equilibrium, the total number of life-years produced by a person with type 1 diabetes can be allocated to successive age intervals from disease onset. Estimates of cumulative survival at attained age have, for each age group at onset, been derived from the Danish data described above. After adjusting for the penalty value 0.033 assigned to Denmark the default values used for maximum mean cumulative are summarized in ESM Table 5. The age interval 65+ years is not shown because it is open-ended with a cumulative survival at 0 by definition. For the same reason age at onset group 65+ years is not shown.

EMS Fig. 2 illustrates the principles of allocating prevalent case numbers to groups by attained age using the data in EMS Table 4 and using New Zealand as an example.

The principle is that each person with type 1 diabetes represents one lived life-year, and the fractions of this life-year that are spent in each interval after onset are estimated as the respective areas under the curve as shown in Figure 2. For each group defined by age at onset, the number of incident cases is multiplied by the fraction surviving for every age interval after onset of the disease, providing the contributions to the number of prevalent

cases by attained age from each of the age at onset groups. For the open-ended oldest attained age interval, the maximum mean duration will vary between countries depending on circumstances. Furthermore, the number of prevalent cases in this age group is obtained by the total number of prevalent cases not accounted for in the younger intervals.

The calculations are further explained in Table 5 using New Zealand as an example (penalty value: 0.041). Note that inconsistencies in numbers occur due to rounding errors.

Standardization of prevalence estimates

For age standardization of the country-specific overall prevalence proportions the WHO Standard Population for the period 2000-2015 was used, with the corresponding percentage distribution by age groups 0-14, 15-39, 40-64, and 65+ years: 26.1%, 39.4%, 26.3%, and 8.2%, respectively.

Sensitivity analyses

ESM Table 7 specifies the profiles of the four sensitivity analyses performed separately with the core model as the reference and with focus on the penalty function, mean disease duration, incidence scaling over age at onset groups, respectively. In one of the analyses, the penalty function was not applied (‘ignoring penalty function’), in another, life expectancy was reduced (‘reduced mean duration’) and followed by two different sets of scaling values of incidence rates from childhood to alder age groups (‘changed incidence scaling 1’ and ‘changed incidence scaling 2’).

ESM Table 9 shows the results of the sensitivity analyses, presented as deviations (in %) from the core model.

Electronic Supplementary Material: References

1. Patterson CC, Karuranga S, Salpea P, et al. Worldwide estimates of incidence, prevalence and mortality of type 1 diabetes in children and adolescents: Results from the International Diabetes Federation Diabetes Atlas, 9th edition. Diabetes Res Clin Pract 2019; 157: 107842.

2. United Nations Department of Economic and Social Affairs (2017) Demography data. Available from https://esa.un.org/unpd/wpp/Download/Standard/Population/. Accessed 25 May 2019

3. The World Bank (2020) World Bank Country and Lending Groups. Available from https://datahelpdesk.worldbank.org/knowledgebase/articles/906519-world-bank-country-and-lending-groups. Accessed 5 October 2020

4. Alemu S, Dessie A, Seid E, Bard E, Lee PT, Trimble ER, et al. Insulin–requiring diabetes in rural Ethiopia: should we reopen the case for malnutrition–related diabetes? Diabetologia. 2009;52(9):1842–5.

5.DIAMOND Project Group. Incidence and trends of childhood Type 1 diabetes worldwide 1990–1999. Diabet Med. 2006;23(8):857–66.

6. Marshall S, Edidin D, Arena VC, Becker DJ, Bunker CH, Gishoma C, et al. Prevalence and incidence of clinically recognized cases of Type 1 diabetes in children and adolescents in Rwanda, Africa. Diabet Med. 2015;32(9):1186–92.

7. Swai AB, Lutale JL, McLarty DG. Prospective study of incidence of juvenile diabetes mellitus over 10 years in Dar es Salaam, Tanzania. BMJ. 1993;306(6892):1570–2.

8. Niar S, Naceur M, Bessahraoui M, Bouchetara A, Zennaki A, Gharnouti M, et al. Épidémiologie du diabète de type 1 de l’enfant dans le département d’Oran (Algérie), de 1975 à 2014. Médecine des Mal Métaboliques. 2015;9(5):529–32.

9. El–Ziny MAE–M, Salem NA–B, El–Hawary AK, Chalaby NM, Elsharkawy AA–E.

Epidemiology of childhood type 1 diabetes mellitus in Nile Delta, northern Egypt – a retrospective study. J Clin Res Pediatr Endocrinol. 2014;6(1):9–15.

10. Kadiki OA, Roaeid RBM. Incidence of type 1 diabetes in children (0–14 years) in Benghazi Libya (1991–2000). Diabetes Metab. 2002;28(6 pt I):463–7.

11. Elamin A, Ghalib M, Eltayeb B, Tuvemo T. High incidence of type 1 diabetes mellitus in Sudanese children, 1991–1995. Ann Saudi Med. 1997;17(4):478–80.

12. Weng J, Zhou Z, Guo L, Zhu D, Ji L, Luo X, Mu Y, Jia W and the T1D China Study Group. Incidence of type 1 diabetes in China, 2010–13: population based study. BMJ. 2018;360:j5295.

13. Onda Y, Sugihara S, Ogata T, Yokoya S, Yokoyama T, Tajima N. Incidence and prevalence of childhood–onset Type 1 diabetes in Japan: the T1D study. Diabet Med. 2017; 34(7):909–15.

14. Kim JH, Lee CG, Lee YA, Yang SW, Shin CH. Increasing incidence of type 1 diabetes among Korean children and adolescents: analysis of data from a nationwide registry in Korea. Pediatr Diabetes. 2016;17(7):519–24.

15. Rakhimova G, Alimova NU, Ryaboshtan A, Waldman B, Ogle G, Ismailov SI. Epidemiological data of type 1 diabetes mellitus in children in Uzbekistan, 1998–2014. Pediatr Diabetes. 2018;19(1):158–65.

16 Balsa A, Zabeen B, Ogle G, Tayyeb S, Kishwar Azad. Incidence estimate of type 1 Diabetes in Youth in Dhaka. In: Endocrine Abstracts. Lisbon; 2017. p. EP428.

17. Jensen ET, Dabelea D, Praveen PA, Anandakumar A, Hockett CW, Isom S, Ong TC, Mohan V, D'Agostino Jr. R, Kahn MG, Hamman RF, Tandon N, Mayer–Davis E. P134 Comparison of the incidence of diabetes in U.S. and Indian youth: an international harmonisation of youth diabetes registries. Pediatric Diabetes 2018; 19(S26): 91.

18. Pishdad GR. Low Incidence of Type 1 Diabetes in Iran. Diabetes Care. 2005;28(4):927–8.

19. Ogle G, Taito R, Besancon S, Sidibe A, Alghassoum W, Majeed N, et al. Establishment of diabetes registers in four under–resourced countries. In: Pediatric Diabetes. 2014. p. 16–48.

20. Lee W, Ooi BC, Thai AC, Loke KY, Tan YT, Rajan U, et al. The Incidence of IDDM in Singapore Children. Singapore Med J. 1998;39(8):359–62.

21. Panamonta O, Thamjaroen J, Panamonta M, Panamonta N, Suesirisawat C. The rising incidence of type 1 diabetes in the northeastern part of Thailand. J Med Assoc Thail. 2011;94(12):1447–50.

22. Navasardyan L V. Epidemiology of type 1 diabetes in children and adolescents in the Republic of Armenia. New Armen Med J. 2014;8(3):89–94.

23. Ahmadov GA, Govender D, Atkinson MA, Sultanova RA, Eubova AA, Wasserfall CH et al. Epidemiology of childhood–onset type 1 diabetes in Azerbaijan: Incidence, clinical features, biochemistry, and HLA–DRB1 status. Diabetes Res Clin Pract. 2018;144:252–259.

24. Skordis N, Efstathiou E, Kyriakides T, Savvidou A, Savva S, Phylactou L, et al. Epidemiology of Type 1 diabetes mellitus in Cyprus: rising incidence at the dawn of the 21st century. Hormones. 2012;11(1):86–93.

25. Amirkhanashvili K, Bikashvili N, Metreveli D, Koplatadze K, Kacharavara L. Epidemiology of The Diabetes Type 1 in Georgian Children Population, 1990–1999 Study. In: 36th Annual EASD Meeting. Diabetologia 2000; 43(suppl 1) A93.

26. Zung A, Blumenfeld O, Shehadeh N, Dally Gottfried O, Tenenbaum Rakover Y, Hershkovitz E, et al. Increase in the incidence of type 1 diabetes in Israeli children following the Second Lebanon War. Pediatr Diabetes. 2012;13(4):326–33.

27. Ajlouni K, Qusous Y, Khawaldeh A, Jaddou H, Batiehah A, Ammari F, et al. Incidence of insulin–dependent diabetes mellitus in Jordanian children aged 0–14 y during 1992–1996. Acta Paediatr. 1999;88(suppl 427):11–3.

28. Shaltout AA, Wake D, Thanaraj TA, Omar DM, Al–AbdulRazzaq D, Channanath A, et al. Incidence of type 1 diabetes has doubled in Kuwaiti children 0–14 years over the last 20 years. Pediatr Diabetes. 2017;18(8):761–6.

29. Soliman AT, Al–Salmi IS, Asfour MG. Epidemiology of childhood insulin–dependent diabetes mellitus in the Sultanate of Oman. Diabet Med. 1996;13(6):582–6.

30. Alyafei F, Soliman A, Alkhalaf F, Sabt A, De Sanctis V, Waseef R, Elsayed N.Al–Zyoud M, Ali M, Rahim A, Ibrahim M. Incidence of type 1 and type 2 diabetes, between 2012–2016, among children and adolescents in Qatar. Acta Biomed. 2018; 89(S5):7–10.

31. Habeb AM, Al–Magamsi MS, Halabi S, Eid IM, Shalaby S, Bakoush O. High incidence of childhood type 1 diabetes in Al–Madinah, North West Saudi Arabia (2004–2009). Pediatr Diabetes. 2011;12(8):676–81.

32. Yeşilkaya E, Cinaz P, Andıran N, Bideci A, Hatun Ş, Sarı E, et al. First report on the nationwide incidence and prevalence of Type 1 diabetes among children in Turkey. Diabet Med. 2017;34(3):405–10.

33. Zalutskaya A, Bornstein SR, Mokhort T, Garmaev D. Did the Chernobyl incident cause an increase in Type 1 diabetes mellitus incidence in children and adolescents? Diabetologia. 2004;47(1):147–8.

34. Patterson CC, Harjutsalo V, Rosenbauer J, Neu A, Cinek O, Skrivarhaug T et al. Trends and cyclical variation in the incidence of childhood type 1 diabetes in 26 European centres in the 25 year period 1989–2013: a multicentre prospective registration study. Diabetologia. 2019;62(3):408–17.

35. Szalecki M, Wysocka–Mincewicz M, Ramotowska A, Mazur A, Lisowicz L, Beń–Skowronek I, Sieniawska J, Klonowska B, Charemska D, Nawrotek J, Jałowiec I, Bossowski A, Jamiołkowska M, Pyrżak B, Miszkurka G, Szypowska A. Epidemiology of type 1 diabetes in Polish children: A multicentre cohort study. Diabetes Metab Res Rev. 2018;34(2):e2962.

36. Serban V, Brink S, Timar B, Sima A, Vlad M, Timar R, et al. An increasing incidence of type 1 diabetes mellitus in Romanian children aged 0 to 17 years. J Pediatr Endocrinol Metab. 2015; 28(3–4):293–8.

37. Shiryaeva TY, Andrianova EA, Suntsov YI. Type 1 diabetes mellitus in children and adolescents of Russian Federation: key epidemiology trends. Diabetes Mellit. 2013;16(3):21–9.

38. Patterson CC, Dahlquist GG, Gyürüs E, Green A, Soltész G. Incidence trends for childhood type 1 diabetes in Europe during 1989–2003 and predicted new cases 2005–20: a multicentre prospective registration study. Lancet. 2009;373(9680):2027–33.

39. Timchenko O, Kozachok GS, Turos EI, Omelchenko EM. The prevalence of diabetes mellitus in children of different regions of Ukraine. Tsitol Genet. 1996;30(6):70–3.

40. Teeäär T, Liivak N, Heilman K, Kool P, Sor R, Paal M, et al. Increasing incidence of childhood–onset type 1 diabetes mellitus among Estonian children in 1999–2006. Time trend analysis 1983–2006. Pediatr Diabetes. 2010;11(2):107–110.

41. Harjutsalo V, Sund R, Knip M, Groop P–H. Incidence of Type 1 Diabetes in Finland. J Am Med Assoc. 2013;310(4):1–12.

42. Green A, Patterson CC. Trends in the incidence of childhood–onset diabetes in Europe 1989–1998. Diabetologia. 2001;44(suppl 3):B3–8.

43. Berhan Y, Waernbaum I, Lind T, Möllsten A, Dahlquist G. Thirty Years of Prospective Nationwide Incidence of Childhood Type 1 Diabetes The Accelerating Increase by Time Tends to Level Off in Sweden. Diabetes. 2011;60(2):577–81.

44. Radosevic B, Bukara–Radujkovic G, Miljkovic V, Pejicic S, Bratina N, Battelino T. The incidence of type 1 diabetes in Republic of Srpska (Bosnia and Herzegovina) and Slovenia in the period 1998–2010. Pediatr Diabetes. 2013;14(4):273–9.

45. Rojnic Putarek N, Ille J, Spehar Uroic A, Skrabic V, Stipancic G, Krnic N, et al. Incidence of type 1 diabetes mellitus in 0 to 14–yr–old children in Croatia – 2004 to 2012 study. Pediatr Diabetes. 2015;16(6):448–453.

46. Mamoulakis, D, Vrouvaki F, Louvari V,. Galanakis E. Incidence of childhood Type 1 diabetes mellitus in Crete. Diabet Med 2018 May 23.[Epub ahead of print]

47. Bruno G, Pagano E, Rossi E, Cataudella S, De Rosa M, Marchesini G, Miccoli R, Vaccaro O, Bonora E. Incidence, prevalence, costs and quality of care of type 1 diabetes in Italy, age 0–29 years: The population–based CINECA–SID ARNO Observatory, 2002–2012. Nutr Metab Cardiovasc Dis. 2016;26(12):1104–11.

48. Formosa N, Calleja N, Torpiano J. Incidence and modes of presentation of childhood type 1 diabetes mellitus in Malta between 2006 and 2010. Pediatr Diabetes. 2012;13(6):484–8.

49. Vukovic R, Jesic MD, Vorgucin I, Stankovic S, Folic N, Milenkovic T, Sajic S, Katanic D, Zivic S, Markovic S, Soldatovic I. First report on the nationwide incidence of type 1 diabetes and ketoacidosis at onset in children in Serbia: a multicenter study. Eur J Pediatr. 2018;177(8):1155–62.

50. Barreiro SC, Rigual MR, Lozano GB, Siguero JPL, Pelegrín BG, Val MPR, Dea MLC. Epidemiology of type 1 diabetes mellitus in children in Spain. An Pediatr (Barc). 2014 Sep;81(3):189.e1–12.

51. Piffaretti C, Mandereau–Bruno L, Guilmin–CreponS, Choleau C, Coutant R, Fosse–Edorh S. Trends in childhood type 1 diabetes incidence in France, 2010–2015. Diabetes Res Clin Pract. 2019;149(1):200–7.

52. Spaans EA, Gusdorf LM, Groenier KH, Brand PL, Veeze HJ, Reeser HM, et al. The incidence of type 1 diabetes is still increasing in the Netherlands, but has stabilised in children under five (Young DUDEs–1). Acta Paediatr. 2015;104(6):626–9.

53. Tull E, Jordan O, Simon L, Laws M, Smith D, Vanterpool H, et al. Incidence of Childhood–Onset IDDM in Black African–Heritage Populations in the Caribbean. Diabetes Care. 1997;20(3):309–10.

54. Peter SA, Johnson R, Taylor C, Hanna A, Roberts P, McNeil P, et al. The incidence and prevalence of type–1 diabetes mellitus. J Natl Med Assoc. 2005;97(2):250–2.

55. Jordan O, Lipton RB, Stupnicka E, Cruickshank KJ, Fraser HS. Incidence of Type I Diabetes in People Under 30 Years of Age in Barbados, West Indies, 1982–1991. 1994;17(5):428–31.

56. Karvonen M, Viik–Kajander M, Moltchanova E, Libman I, LaPorte R, Tuomilehto J. Incidence of childhood type 1 diabetes worldwide. Diabetes Mondiale (DiaMond) Project Group. Diabetes Care. 2000;23(10):1516–26.

57. Gómez–Díaz RA, Pérez–Pérez G, Hernández–Cuesta IT, Rodríguez–García JDC, Guerrero–López R, Aguilar–Salinas CA, et al. Incidence of type 1 diabetes in Mexico: data from an institutional register 2000–2010. Diabetes Care. 2012;35(11):e77.

58. Duarte Gómez E, Gregory GA, Castrati Nostas M, Middlehurst AC, Jenkins AJ, Ogle GD, et al. Incidence and Mortality Rates and Clinical Characteristics of Type 1 Diabetes among Children and Young Adults in Cochabamba, Bolivia. J Diabetes Res. 2017;2017:1–8.

59. Negrato CA, Lauris JRP, Saggioro IB, Corradini MCM, Borges PR, Crês MC, et al. Increasing incidence of type 1 diabetes between 1986 and 2015 in Bauru, Brazil. Diabetes Res Clin Pract. 2017;127:198–204.

60. Garfias C, Pinochet C, Borzutzky A, Cerda J, Ugarte F, Martin Y, et al. Rising of type 1

diabetes mellitus incidence in Chilean children between 2006 and 2014 Final Results. In: 55th Annual ESPE. Paris: ESPE Abstracts; 2016. p. 1–227.

61. Fox DA, Islam N, Sutherland J, Reimer K, Amed S. Type 1 diabetes incidence and prevalence trends in a cohort of Canadian children and youth. Pediatr Diabetes 2018; 19(3):501–505.

62. Mayer–Davis E, Lawrence J, Dabelea D, Divers J, Dolan L, Imperatore G, et al. Incidence Trends of Type 1 and Type 2 Diabetes among Youths, 2002–2012. N Engl J Med. 2017;376(15) 1419–29.

63. Haynes A, Bulsara MK, Bower C, Jones TW, Davis EA. Regular peaks and troughs in the Australian incidence of childhood type 1 diabetes mellitus (2000–2011). Diabetologia. 2015;58(11):2513–6.

64. Derraik JG, Reed PW, Jefferies C, Cutfield SW, Hofman PL, Cutfield WS. Increasing incidence and age at diagnosis among children with type 1 diabetes mellitus over a 20–year period in Auckland (New Zealand). PLoS One. 2012;7(2):e32640.

65. Ogle G, Morrison MK, Silink M, Taito RS. Incidence and prevalence of diabetes in children aged <15 yr in Fiji, 2001–2012. Pediatr Diabetes. 2016;17(3):222–6.

66. Ogle G, Lesley J, Sine P, McMaster P. Type 1 diabetes mellitus in children in Papua New Guinea. P N G Med J. 2001;(3–4):96–100.

67. Green A, Sortso C, Jensen PB, Emneus M Incidence, morbidity, mortality, and prevalence of diabetes in Denmark, 2000-2011: results from the Diabetes Impact Study 2013. Clin Epidemiol.. 2015;7:421-30.

68. United Nations Inter-agency Group for Child Mortality Estimation (2019) Child Mortality Estimates. Available from https://childmortality.org/ Accessed 26 November 2019

69. Livingstone SJ, Levin D, Looker HC, et al. Estimated life expectancy in a Scottish cohort with type 1 diabetes, 2008-2010. JAMA. 2015;313:37-44.

EMS Table 1: Country-specific background data

Income level: LIC: Low-income country; LMIC: Lower middle income country; UMIC: Upper middle income country; HIC: High income country)

Childhood mortality: Mortality rate under age 5 years (per 1000)

Penalty (Pen): A value assigned to a country to adjust for health and living standards. Pen = CM/130

Major area, region, country or area	Income level	Childhood Mortality	Penalty	Applied incidence rate, both sexes, age group 0-14 yr (per 100,000)		
				Rate	IDF reference	Country used for extrapolation
AFRICA						
Eastern Africa						
Burundi	LIC	61.2	0.471	1.2		Rwanda
Comoros	LIC	69.0	0.531	1.4		Mauritius
Djibouti	LMIC	61.7	0.475	0.3		Ethiopia
Eritrea	LIC	43.1	0.332	0.3		Ethiopia
Ethiopia	LIC	58.5	0.450	0.3	[4]	
Kenya	LIC	45.6	0.351	0.9		United Republic of Tanzania
Madagascar	LIC	44.2	0.340	0.9		United Republic of Tanzania
Malawi	LIC	55.4	0.426	0.9		United Republic of Tanzania
Mauritius	UMIC	13.1	0.101	1.4	[5]	
Mozambique	LIC	72.4	0.557	0.9		United Republic of Tanzania
Rwanda	LIC	37.9	0.292	1.2	[6]	
Seychelles	UMIC	14.2	0.109	1.4		Mauritius
Somalia	LIC	127.2	0.978	0.3		Ethiopia
South Sudan	LMIC	96.4	0.742	0.9		United Republic of Tanzania
Uganda	LIC	49.0	0.377	1.2		Rwanda
United Republic of Tanzania	LIC	54.0	0.415	0.9	[7]	
Zambia	LMIC	60.0	0.462	0.9		United Republic of Tanzania
Zimbabwe	LIC	50.3	0.387	0.9		United Republic of Tanzania
Middle Africa						
Angola	UMIC	81.1	0.624	1.2		Rwanda
Cameroon	LMIC	84.0	0.646	1.2		Rwanda
Central African Republic	LIC	121.5	0.935	1.2		Rwanda
Chad	LIC	123.2	0.948	1.2		Rwanda
Congo	LMIC	47.5	0.365	1.2		Rwanda
Democratic Republic of the Congo	LIC	91.1	0.701	1.2		Rwanda
Equatorial Guinea	HIC	89.6	0.689	1.2		Rwanda
Gabon	UMIC	48.3	0.372	1.2		Rwanda
Sao Tome and Principe	LMIC	32.4	0.249	1.2		Rwanda

Northern Africa						
Algeria	UMIC	24.0	0.185	24.3	[8]	
Egypt	LMIC	22.1	0.170	3.1	[9]	
Libya	UMIC	12.4	0.095	9.0	[10]	
Morocco	LMIC	23.3	0.179	24.3		Algeria
Sudan	LMIC	63.2	0.486	10.1	[11]	
Tunisia	LMIC	13.0	0.100	7.3	[5]	
Southern Africa						
Botswana	UMIC	37.6	0.289	1.2		Rwanda
Lesotho	LMIC	85.9	0.661	0.9		United Republic of Tanzania
Namibia	UMIC	44.2	0.340	1.2		Rwanda
South Africa	UMIC	37.1	0.285	0.9		United Republic of Tanzania
Eswatini	LMIC	53.9	0.415	0.9		United Republic of Tanzania
Western Africa						
Benin	LIC	98.3	0.756	1.2		Rwanda
Burkina Faso	LIC	81.2	0.625	1.2		Rwanda
Cabo Verde	LMIC	17.4	0.134	1.2		Rwanda
Côte d'Ivoire	LMIC	88.8	0.683	1.2		Rwanda
Gambia	LIC	63.6	0.489	1.2		Rwanda
Ghana	LIC	49.3	0.379	1.2		Rwanda
Guinea	LIC	85.7	0.659	1.2		Rwanda
Guinea-Bissau	LIC	84.2	0.648	1.2		Rwanda
Liberia	LIC	74.7	0.575	1.2		Rwanda
Mali	LIC	106.0	0.815	1.2		Rwanda
Mauritania	LMIC	79.0	0.608	1.2		Rwanda
Niger	LIC	84.5	0.650	1.2		Rwanda
Nigeria	LMIC	100.2	0.771	1.2		Rwanda
Senegal	LMIC	45.4	0.349	1.2		Rwanda
Sierra Leone	LIC	110.5	0.850	1.2		Rwanda
Togo	LIC	72.9	0.561	1.2		Rwanda

Major area, region, country or area	Income level	Childhood Mortality	Penalty	Applied incidence rate, both sexes, age group 0-14 yr (per 100,000) Rate	IDF reference	Country used for extrapolation
ASIA						
Eastern Asia						
China	LMIC	9.3	0.072	1.9	[12]	
Democratic People's Republic of Korea	LIC	19.0	0.146	3.2		Republic of Korea
Japan	HIC	2.6	0.020	2.2	[13]	
Mongolia	LMIC	17.2	0.132	1.9		China
Republic of Korea	HIC	3.3	0.025	3.2	[14]	
South-Central Asia						
Central Asia						
Kazakhstan	UMIC	10.0	0.077	1.9		China
Kyrgyzstan	LIC	20.0	0.154	1.9		China
Tajikistan	LIC	33.6	0.258	2.6		Uzbekistan
Turkmenistan	UMIC	47.3	0.364	11.0		Turkey
Uzbekistan	LMIC	22.5	0.173	2.6	[15]	
Southern Asia						
Afghanistan	LIC	67.9	0.522	2.6		Uzbekistan
Bangladesh	LIC	32.4	0.249	1.1	[16]	
Bhutan	LMIC	30.8	0.237	4.3		India
India	LMIC	39.4	0.303	4.3	[17]	
Iran (Islamic Republic of)	UMIC	14.9	0.115	3.7	[18]	
Maldives	LMIC	7.9	0.061	3.2	[19]	
Nepal	LIC	33.7	0.259	4.3		India
Pakistan	LMIC	74.9	0.576	0.5	[5]	
Sri Lanka	LMIC	8.8	0.068	4.3		India
South-Eastern Asia						
Brunei Darussalam	HIC	10.5	0.081	1.0		Thailand
Cambodia	LIC	29.2	0.225	1.0		Thailand
Indonesia	LMIC	25.4	0.195	1.0		Thailand
Lao People's Democratic Republic	LIC	63.4	0.488	1.0		Thailand
Malaysia	UMIC	7.9	0.061	1.0		Thailand
Myanmar	LIC	48.6	0.374	1.0		Thailand
Philippines	LMIC	28.1	0.216	1.0		Thailand
Singapore	HIC	2.8	0.022	2.4	[20]	
Thailand	LMIC	9.5	0.073	1.0	[21]	

Major area, region, country or area	Income level	Childhood Mortality	Penalty	Applied incidence rate, both sexes, age group 0-14 yr (per 100,000) Rate	IDF reference	Country used for extrapolation
Timor-Leste	LMIC	47.6	0.366	1.0		Thailand
Viet Nam	LMIC	20.9	0.161	1.0		Thailand
Western Asia						
Armenia	LMIC	12.6	0.097	7.0	[22]	
Azerbaijan	LMIC	23.0	0.177	7.1	[23]	
Bahrain	HIC	7.3	0.056	2.5		Oman
Cyprus	HIC	2.7	0.021	14.4	[24]	
Georgia	LMIC	10.8	0.083	4.6	[25]	
Iraq	LMIC	30.4	0.234	3.2		Jordan
Israel	HIC	3.6	0.028	14.9	[26]	
Jordan	LMIC	17.0	0.131	3.2	[27]	
Kuwait	HIC	8.1	0.062	41.7	[28]	
Lebanon	UMIC	7.8	0.060	3.2		Jordan
Oman	HIC	11.3	0.087	2.5	[29]	
Qatar	HIC	7.6	0.058	28.4	[30]	
Saudi Arabia	HIC	7.4	0.057	31.4	[31]	
Syrian Arab Republic	LMIC	17.0	0.131	3.2		Jordan
Turkey	UMIC	11.6	0.089	11.0	[32]	
United Arab Emirates	HIC	9.1	0.070	2.5		Oman
Yemen	LMIC	55.4	0.426	2.5		Oman

Major area, region, country or area	Income level	Childhood Mortality	Penalty	Applied incidence rate, both sexes, age group 0-14 yr (per 100,000)		
				Rate	IDF reference	Country used for extrapolation
EUROPE						
Eastern Europe						
Belarus	UMIC	3.7	0.028	5.6	[33]	
Bulgaria	UMIC	7.5	0.058	9.4	[5]	
Czechia	HIC	3.3	0.025	21.8	[34]	
Hungary	UMIC	4.5	0.035	20.1	[34]	
Poland	HIC	4.7	0.036	18.8	[35]	
Republic of Moldova	LMIC	15.5	0.119	7.7		Romania
Romania	UMIC	7.8	0.060	7.7	[36]	
Russian Federation	HIC	7.6	0.058	12.4	[37]	
Slovakia	HIC	5.6	0.043	13.6	[38]	
Ukraine	LMIC	8.8	0.068	7.9	[39]	
Northern Europe						
Denmark	HIC	4.3	0.033	27.0	[34]	
Estonia	HIC	2.7	0.021	17.1	[40]	
Finland	HIC	2.3	0.018	62.3	[41]	

Iceland	HIC	2.1	0.016	14.7	[42]	
Ireland	HIC	3.5	0.027	27.5	[34]	
Latvia	HIC	4.2	0.032	7.5	[42]	
Lithuania	HIC	4.3	0.033	19.9	[34]	
Norway	HIC	2.6	0.020	33.6	[34]	
Sweden	HIC	2.8	0.022	43.2	[43]	
United Kingdom	HIC	4.3	0.033	29.4	[34]	
Southern Europe						
Albania	LMIC	8.8	0.068	7.7		TFYR Macedonia
Andorra	HIC	3.3	0.025	17.7		Spain
Bosnia and Herzegovina	UMIC	5.7	0.044	8.2	[44]	
Croatia	HIC	4.6	0.035	17.2	[45]	
Greece	HIC	5.3	0.041	15.8	[46]	
Italy	HIC	3.4	0.026	16.2	[47]	
Malta	HIC	6.4	0.049	21.9	[48]	
Montenegro	UMIC	3.5	0.027	18.5	[34]	
Portugal	HIC	3.7	0.028	13.2	[42]	
San Marino	HIC	2.2	0.017	16.2		Italy
Serbia	UMIC	5.7	0.044	14.5	[49]	
Slovenia	HIC	2.1	0.016	16.3	[34]	
Spain	HIC	3.1	0.024	17.7	[50]	
TFYR Macedonia	UMIC	13.7	0.105	7.7	[34]	
Western Europe						
Austria	HIC	3.6	0.028	19.7	[34]	
Belgium	HIC	3.8	0.029	18.1	[34]	
France	HIC	4.2	0.032	18.9	[51]	
Germany	HIC	3.7	0.028	24.3	[34]	
Luxembourg	HIC	2.6	0.020	18.6	[34]	
Monaco	HIC	3.3	0.025	18.9		France
Netherlands	HIC	3.9	0.030	21.2	[52]	
Switzerland	HIC	4.2	0.032	13.4	[34]	

Major area, region, country or area	Income level	Childhood Mortality	Penalty	Applied incidence rate, both sexes, age group 0-14 yr (per 100,000)		
				Rate	IDF reference	Country used for extrapolation
LATIN AMERICA AND THE CARIBBEAN						
Caribbean						
Antigua and Barbuda	HIC	7.4	0.057	3.5	[53]	
Bahamas	HIC	7.2	0.055	10.1	[54]	
Barbados	HIC	12.4	0.095	5.0	[55]	
Cuba	UMIC	5.4	0.042	2.3	[5]	
Dominica	UMIC	34.0	0.262	5.7	[56]	
Dominican Republic	UMIC	29.9	0.230	0.5	[5]	
Grenada	UMIC	16.7	0.128	5.0		Barbados
Haiti	LIC	71.7	0.552	0.5		Dominican Republic
Jamaica	UMIC	15.2	0.117	2.3		Cuba
Saint Kitts and Nevis	HIC	13.7	0.105	5.0		Barbados
Saint Lucia	UMIC	16.6	0.128	5.0		Barbados
Saint Vincent and the Grenadines	UMIC	16.2	0.125	5.0		Barbados
Trinidad and Tobago	HIC	26.1	0.201	5.0		Barbados
Central America						
Belize	LMIC	14.2	0.109	6.2		Mexico
Costa Rica	UMIC	9.0	0.069	1.3		Colombia
El Salvador	LMIC	14.5	0.112	6.2		Mexico
Guatemala	LMIC	27.6	0.212	6.2		Mexico
Honduras	LMIC	18.2	0.140	6.2		Mexico
Mexico	UMIC	13.4	0.103	6.2	[57]	
Nicaragua	LMIC	17.2	0.132	6.2		Mexico
Panama	UMIC	16.1	0.124	1.3		Colombia
South America						
Argentina	UMIC	10.4	0.080	6.8	[5]	
Bolivia (Plurinational State of)	LMIC	34.9	0.268	2.2	[58]	
Brazil	UMIC	14.8	0.114	16.3	[59]	
Chile	HIC	7.4	0.057	13.9	[60]	
Colombia	UMIC	14.7	0.113	1.3	[5]	
Ecuador	LMIC	14.5	0.112	1.3		Colombia

Guyana	LMIC	31.3	0.241	0.1		Venezuela (Bolivarian Republic of)
Paraguay	LMIC	21.0	0.162	0.9	[5]	
Peru	UMIC	15.0	0.115	0.5	[5]	
Suriname	UMIC	19.6	0.151	0.1		Venezuela (Bolivarian Republic of)
Uruguay	HIC	8.2	0.063	8.3	[5]	
Venezuela (Bolivarian Republic of)	UMIC	30.9	0.238	0.1	[5]	
Major area, region, country or area	Income level	Childhood Mortality	Penalty	Applied incidence rate, both sexes, age group 0-14 yr (per 100,000)		
				Rate	IDF reference	Country used for extrapolation
NORTHERN AMERICA						
Canada	HIC	5.1	0.039	29.9	[61]	
United States of America	HIC	6.6	0.051	23.8	[62]	
OCEANIA						
Australia/New Zealand						
Australia	HIC	3.5	0.027	23.6	[63]	
New Zealand	HIC	5.3	0.041	22.4	[64]	
Melanesia						
Fiji	UMIC	25.3	0.195	0.9	[65]	
Papua New Guinea	LMIC	53.4	0.411	0.1	[66]	
Solomon Islands	LMIC	20.6	0.158	0.1		Papua New Guinea
Vanuatu	LMIC	26.9	0.207	0.9		Fiji
Micronesia						
Kiribati	LMIC	54.6	0.420	0.9		Fiji
Marshall Islands	LMIC	34.0	0.262	0.1		Papua New Guinea
Micronesia (Fed. States of)	LMIC	32.2	0.248	0.1		Papua New Guinea
Nauru	LMIC	33.0	0.254	0.1		Papua New Guinea
Palau	UMIC	15.3	0.118	0.1		Papua New Guinea
Polynesia						
Cook Islands	HIC	25.0	0.192	0.9		Fiji
Niue	UMIC	21.5	0.165	0.9		Fiji
Samoa	UMIC	16.5	0.127	0.9		Fiji
Tonga	LMIC	16.0	0.123	0.9		Fiji
Tuvalu	UMIC	24.9	0.192	0.9		Fiji

ESM Table 2. Scaling age-specific incidence rates relative to the incidence rate for children aged 0-14 years

Age at onset group (years)	Incidence rate (per 100,000	Scaling factor
0-14	33.8	1.00 (Reference)
15-39	17.2	0.50
40-64	9.8	0.30
65+	8.0	0.25

ESM Table 3. Maximum mean remaining life-years by age group at onset

Age group	Mean age at onset[a]	Estimated remaining life-years from onset[b]	Maximum mean remaining life-years from onset[c]
0-14	9	58.4	60.4
15-39	26	42.0	43.4
40-64	52	20.4	21.1
65+	74	8.2	8.5

[a] Based on incident cases of type 1 diabetes, Denmark, 2015-2017
[b] Based on mortality rates in patients with type 1 diabetes, Denmark, 2015-2017
[c] Adjusted for a penalty value (Pen) at 0.033 assigned to Denmark

ESM Table 4. Estimation of prevalence by age at onset. Example: New Zealand

Age group (years)	Population size (in 1,000s)	Incidence rate (per 100,000)	Incident cases (in 1,000s)	Remaining life-years[a]	Prevalent cases (in 1,000s) by age at onset[b]
0-14	930	22.40	930 x 22.40 = 0.208	60.4 x (1-0.041)=57.9	0.208 x 57.9 = 12.071
15-39	1,559	11.20	1,559 x 11.20 = 0.175	43.4 x (1-0.041)=41.6	0.175 x 41.6 = 7.270
40-64	1,495	6.72	1,495 x 6.72 = 0.100	21.1 x (1-0.041)=20.2	0.100 x 20.2 = 2.034
65+	721	5.60	721 x 5.60 = 0.040	8.5 x (1-0.041)=8.2	0.040 x 8.2 = 0.329
Total	4,706	NA	0.524[b]	NA	21.705

[a] Applying a penalty value (Pen) = 0.041 assigned to New Zealand
[b] Minor inconsistencies in calculations for a country may occur due to rounding
NA: Not applicable

ESM Table 5. Default values for maximum cumulative survival from onset to the attained age[a]

	Maximum cumulative survival from onset to attained age		
Age group at onset (years)[a]	15 years	40 years	65 years
0-14 years	1.00	0.97	0.78
15-39 years	NA	0.98	0.83
40-64 years	NA	NA	0.88

NA: Not applicable

[a] Ages of 65 years or more are not shown because the age interval is open-ended with a cumulative survival ai 0 per definition

ESM Table 6. Estimation of prevalent cases by attained age. Example: New Zealand[a]. See also ESM Figure 2 for illustration.
A, B, C and D refer to estimated prevalent cases for attained age groups 0-14, 15-39, 40-64 and 65+ years, respectively.
The subscripts 0-14, 15-39, 40-64 and 65+ refer to age at onset groups 0-14, 15-39, 40-64 and 65+ years, respectively.

Age at onset (years)	Incident cases[b] (in 1,000s)	Attained age group 0-14	Attained age group 15-39	Attained age group 40-64	Attained age group 65+	Total[c] (in 1,000s)
0-14	0.208	A_{0-14} = 0.208*(15-9) *(1.00+(1*0.959))/2 = 1.225	B_{0-14} = 0.208*25*0.959 *(1.00+0.97)/2 = 4.921	C_{0-14} = 0.208*25*0.959*(0.97+0.78)/2 = 4.372	D_{0-14} = 12.071-1.225-4.921-4.372 = 1.553	12.071
15-39	0.175	NA	B_{15-39} = 0.175*(40-26) *(1.00+(0.959*0.98))/2 = 2.372	C_{15-39} = 0.175*25*0.959*(0.98+0.83)/2 = 3.790	D_{15-39} = 7.270-2.372-3.790 = 1.109	7.270
40-64	0.100	NA	NA	C_{40-64} = 0.100*(65-52) *(1.00+(0.959*0.88))/2 = 1.205	D_{40-64} = 2.034 – 1.205 = 0.829	2.034
65+	0.040	NA	NA	NA	D_{65+} = 0.329[c]	0.329
Total	0.524	1.225	7.293	9.366	3.820	21.705

[a] Applying a penalty value (Pen) = 0.041 assigned to New Zealand, corresponding to an adjustment factor of 1 - 0.041 = 0.959
[b] See ESM Table 3, column 4
[c] See ESM Table 3, column 6
NA: Not applicable

ESM Table 7. Specification of sensitivity analyses, showing the core model and alternative models. Changes in parameters from the core model are given in bold characters

	AGE	CORE MODEL	Ignoring penalty function	Reduced mean duration	Changed incidence scaling 1	Changed incidence scaling 2
Incidence rates relative to age 0-14	Age 0-14	(1)	(1)	(1)	(1)	(1)
	Age 15-39	0.50	0.50	0.50	0.30	0.50
	Age 40-64	0.30	0.30	0.30	0.25	0.50
	Age 65+	0.25	0.25	0.25	0.125	0.50
Mean maximum duration from onset (years)	Age 0-14	60.4	60.4	45.0	60.4	60.4
	Age 15-39	43.4	43.4	30.0	43.4	43.4
	Age 40-64	21.1	21.1	15.0	21.1	21.1
	Age 65+	8.5	8.5	3.0	8.5	8.5
Mean maximum duration to end of interval (years)	Age 0-14	6.0	6.0	4.0	6.0	6.0
	Age 0-39	23.0	23.0	15.0	23.0	23.0
	Age 0-64	35.5	35.5	20.0	35.5	35.5
Penalty function applied		Yes	No	Yes	Yes	Yes

ESM Table 8. Country-specific estimates of numbers of incident and prevalent cases of type 1 diabetes 2017 by age-group

Major area, region, country or area	Incident numbers (in 1000s) 2017 by age groups					Prevalent numbers (in 1000s) 2017 by attained age groups				
	0-14	15-39	40-64	65+	Total	0-14	15-39	40-64	65+	Total
WORLD	97.58	82.23	40.83	14.08	234.71	541.18	3,107.77	3,905.22	1,450.44	9,004.61
AFRICA	12.80	7.53	2.33	0.48	23.13	62.88	289.72	314.37	88.63	755.59
Eastern Africa	1.40	0.65	0.13	0.02	2.21	6.59	26.91	27.16	5.91	66.56
Burundi	0.06	0.03	0.01	<0.01	0.09	0.27	1.04	1.03	0.18	2.52
Comoros	<0.01	<0.01	<0.01	<0.01	0.01	0.02	0.08	0.08	0.01	0.18
Djibouti	<0.01	<0.01	<0.01	<0.01	<0.01	<0.01	0.02	0.02	<0.01	0.05
Eritrea	0.01	<0.01	<0.01	<0.01	0.01	0.03	0.14	0.15	0.04	0.36
Ethiopia	0.13	0.07	0.01	<0.01	0.21	0.59	2.43	2.48	0.46	5.98
Kenya	0.18	0.09	0.02	<0.01	0.30	0.90	3.98	4.16	1.01	10.05
Madagascar	0.09	0.05	0.01	<0.01	0.15	0.47	2.07	2.17	0.55	5.25
Malawi	0.07	0.03	0.01	<0.01	0.12	0.35	1.42	1.43	0.29	3.49
Mauritius	<0.01	<0.01	<0.01	<0.01	0.01	0.02	0.12	0.15	0.06	0.34
Mozambique	0.12	0.05	0.01	<0.01	0.18	0.52	1.82	1.73	0.23	4.30
Rwanda	0.06	0.03	0.01	<0.01	0.10	0.30	1.39	1.47	0.40	3.56
Seychelles	<0.01	<0.01	<0.01	<0.01	<0.01	<0.01	0.01	0.01	<0.01	0.03
Somalia	0.02	0.01	<0.01	<0.01	0.03	0.03	0.01	<0.01	<0.01	0.04
South Sudan	0.05	0.02	0.01	<0.01	0.08	0.18	0.54	0.30	<0.01	1.02
Uganda	0.25	0.10	0.02	<0.01	0.37	1.19	4.89	4.93	1.17	12.18
United Republic of Tanzania	0.23	0.10	0.02	<0.01	0.36	1.10	4.43	4.48	0.97	10.97
Zambia	0.07	0.03	0.01	<0.01	0.11	0.32	1.25	1.24	0.23	3.04
Zimbabwe	0.06	0.03	0.01	<0.01	0.10	0.30	1.29	1.33	0.30	3.20
Middle Africa	0.89	0.37	0.08	0.01	1.36	3.50	10.17	7.70	0.64	22.01
Angola	0.17	0.07	0.01	<0.01	0.25	0.69	2.19	1.94	0.19	5.01
Cameroon	0.12	0.06	0.01	<0.01	0.20	0.50	1.62	1.39	0.11	3.63
Central African Republic	0.02	0.01	<0.01	<0.01	0.04	0.08	0.05	<0.01	<0.01	0.13
Chad	0.08	0.03	0.01	<0.01	0.13	0.27	0.08	0.01	<0.01	0.35
Congo	0.03	0.01	<0.01	<0.01	0.04	0.13	0.55	0.57	0.14	1.40
Democratic Republic of the Congo	0.45	0.18	0.04	0.01	0.68	1.76	5.37	3.51	0.14	10.78
Equatorial Guinea	0.01	<0.01	<0.01	<0.01	0.01	0.02	0.08	0.05	<0.01	0.16
Gabon	0.01	0.01	<0.01	<0.01	0.02	0.04	0.19	0.21	0.05	0.49
Sao Tome and Principe	<0.01	<0.01	<0.01	<0.01	<0.01	0.01	0.03	0.03	0.01	0.07

Northern Africa	8.37	5.52	1.88	0.40	16.16	44.23	225.13	257.41	78.97	605.74
Algeria	2.94	2.06	0.71	0.16	5.86	16.01	85.01	98.37	31.62	231.01
Egypt	1.01	0.61	0.19	0.04	1.85	5.56	28.41	31.96	10.37	76.29
Libya	0.16	0.12	0.04	0.01	0.33	0.92	5.23	6.22	2.16	14.54
Morocco	2.38	1.74	0.67	0.15	4.94	12.99	70.12	82.59	26.98	192.68
Sudan	1.67	0.81	0.20	0.04	2.72	7.59	29.69	30.07	4.90	72.25
Tunisia	0.20	0.17	0.07	0.02	0.46	1.15	6.67	8.20	2.95	18.97
Southern Africa	0.18	0.13	0.04	0.01	0.36	0.91	4.60	5.17	1.39	12.07
Botswana	0.01	0.01	<0.01	<0.01	0.02	0.04	0.22	0.25	0.07	0.59
Lesotho	0.01	<0.01	<0.01	<0.01	0.01	0.03	0.10	0.08	0.01	0.22
Namibia	0.01	0.01	<0.01	<0.01	0.02	0.06	0.26	0.27	0.07	0.66
South Africa	0.15	0.11	0.03	0.01	0.30	0.76	3.92	4.46	1.23	10.37
Eswatini	<0.01	<0.01	<0.01	<0.01	0.01	0.02	0.10	0.10	0.02	0.24
Western Africa	1.95	0.86	0.20	0.03	3.04	7.65	22.92	16.92	1.72	49.21
Benin	0.06	0.03	0.01	<0.01	0.09	0.21	0.60	0.34	<0.01	1.15
Burkina Faso	0.10	0.05	0.01	<0.01	0.16	0.43	1.39	1.23	0.12	3.17
Cabo Verde	<0.01	<0.01	<0.01	<0.01	<0.01	0.01	0.06	0.07	0.02	0.17
Côte d'Ivoire	0.12	0.06	0.01	<0.01	0.20	0.49	1.49	1.21	0.06	3.26
Gambia	0.01	<0.01	<0.01	<0.01	0.02	0.05	0.20	0.19	0.03	0.47
Ghana	0.13	0.07	0.02	<0.01	0.22	0.65	2.83	2.98	0.69	7.15
Guinea	0.06	0.03	0.01	<0.01	0.10	0.26	0.82	0.69	0.05	1.83
Guinea-Bissau	0.01	<0.01	<0.01	<0.01	0.01	0.04	0.12	0.11	0.01	0.27
Liberia	0.02	0.01	<0.01	<0.01	0.04	0.10	0.36	0.34	0.04	0.84
Mali	0.11	0.04	0.01	<0.01	0.16	0.38	0.81	0.36	<0.01	1.55
Mauritania	0.02	0.01	<0.01	<0.01	0.03	0.09	0.31	0.28	0.03	0.71
Niger	0.13	0.04	0.01	<0.01	0.19	0.52	1.53	1.32	0.11	3.48
Nigeria	1.01	0.44	0.10	0.02	1.57	3.72	9.78	5.33	0.03	18.85
Senegal	0.08	0.04	0.01	<0.01	0.13	0.40	1.74	1.80	0.45	4.40
Sierra Leone	0.04	0.02	<0.01	<0.01	0.06	0.13	0.26	0.09	<0.01	0.48
Togo	0.04	0.02	<0.01	<0.01	0.06	0.17	0.61	0.58	0.07	1.43
	Incident numbers (in 1000s) 2017 by age groups					Prevalent numbers (in 1000s) 2017 by attained age groups				
Major area, region, country or area	0-14	15-39	40-64	65+	Total	0-14	15-39	40-64	65+	Total
ASIA	33.56	27.20	11.16	2.47	74.39	179.89	990.31	1,195.99	387.31	2,753.51
Eastern Asia	5.55	5.61	3.50	0.99	15.64	32.15	202.34	272.67	108.49	615.66
China	4.78	4.80	2.94	0.72	13.24	27.68	173.56	232.72	91.03	524.99

Democratic People's Republic of Korea	0.17	0.15	0.08	0.02	0.42	0.93	5.43	6.92	2.46	15.74
Japan	0.36	0.37	0.28	0.19	1.20	2.15	13.82	19.40	9.24	44.61
Mongolia	0.02	0.01	<0.01	<0.01	0.03	0.10	0.53	0.62	0.21	1.47
Republic of Korea	0.22	0.27	0.19	0.06	0.74	1.30	9.00	13.00	5.55	28.85
Central Asia	0.64	0.45	0.15	0.03	1.27	3.43	17.85	20.53	6.25	48.06
Kazakhstan	0.10	0.07	0.03	0.01	0.20	0.56	3.10	3.68	1.34	8.69
Kyrgyzstan	0.04	0.02	0.01	<0.01	0.07	0.20	1.07	1.23	0.40	2.91
Tajikistan	0.08	0.05	0.01	<0.01	0.15	0.43	2.08	2.29	0.66	5.46
Turkmenistan	0.20	0.13	0.04	0.01	0.38	0.96	4.57	5.07	1.20	11.80
Uzbekistan	0.23	0.18	0.06	0.01	0.48	1.27	7.03	8.26	2.65	19.21
Southern Asia	18.59	14.03	4.78	0.98	38.39	94.78	486.25	558.22	152.44	1,291.69
Afghanistan	0.40	0.19	0.04	0.01	0.63	1.77	6.63	6.48	0.94	15.82
Bangladesh	0.52	0.40	0.12	0.02	1.07	2.72	14.48	16.69	4.85	38.73
Bhutan	0.01	0.01	<0.01	<0.01	0.02	0.05	0.27	0.32	0.09	0.73
India	16.00	12.10	4.18	0.86	33.14	81.45	417.13	478.55	128.93	1,106.05
Iran (Islamic Republic of)	0.71	0.69	0.23	0.04	1.67	4.02	24.51	30.17	10.31	69.01
Maldives	<0.01	<0.01	<0.01	<0.01	0.01	0.02	0.12	0.15	0.05	0.35
Nepal	0.39	0.27	0.08	0.02	0.75	2.03	10.36	11.66	3.36	27.42
Pakistan	0.34	0.21	0.06	0.01	0.62	1.46	5.62	5.43	0.60	13.11
Sri Lanka	0.22	0.16	0.08	0.02	0.48	1.25	7.12	8.76	3.32	20.45
South-Eastern Asia	1.66	1.29	0.52	0.10	3.57	8.99	49.00	58.54	18.90	135.44
Brunei Darussalam	<0.01	<0.01	<0.01	<0.01	<0.01	0.01	0.03	0.04	0.02	0.10
Cambodia	0.05	0.03	0.01	<0.01	0.09	0.26	1.35	1.53	0.46	3.60
Indonesia	0.70	0.52	0.20	0.03	1.46	3.79	20.40	24.08	7.66	55.93
Lao People's Democratic Republic	0.02	0.01	<0.01	<0.01	0.04	0.10	0.43	0.45	0.07	1.05
Malaysia	0.07	0.07	0.02	<0.01	0.17	0.43	2.65	3.27	1.18	7.53
Myanmar	0.14	0.11	0.04	0.01	0.29	0.68	3.34	3.82	0.88	8.72
Philippines	0.32	0.21	0.07	0.01	0.61	1.73	8.83	10.01	3.07	23.64
Singapore	0.02	0.02	0.02	<0.01	0.06	0.12	0.81	1.14	0.48	2.55
Thailand	0.12	0.12	0.07	0.02	0.32	0.67	4.19	5.65	2.23	12.74
Timor-Leste	0.01	<0.01	<0.01	<0.01	0.01	0.03	0.11	0.12	0.03	0.28
Viet Nam	0.21	0.19	0.08	0.02	0.50	1.18	6.85	8.44	2.83	19.30
Western Asia	7.11	5.82	2.20	0.37	15.51	40.54	234.87	286.03	101.23	662.67
Armenia	0.04	0.04	0.02	0.01	0.10	0.23	1.43	1.83	0.68	4.17
Azerbaijan	0.16	0.14	0.06	0.01	0.38	0.89	5.11	6.29	2.06	14.35
Bahrain	0.01	0.01	<0.01	<0.01	0.02	0.04	0.30	0.39	0.14	0.88

Cyprus	0.03	0.03	0.02	0.01	0.08	0.17	1.15	1.54	0.62	3.48
Georgia	0.03	0.03	0.02	0.01	0.09	0.20	1.18	1.52	0.60	3.49
Iraq	0.49	0.25	0.06	0.01	0.81	2.62	12.36	13.21	3.93	32.12
Israel	0.35	0.22	0.09	0.04	0.69	2.04	11.25	13.27	5.14	31.70
Jordan	0.11	0.06	0.02	<0.01	0.20	0.62	3.19	3.57	1.19	8.57
Kuwait	0.36	0.38	0.17	0.01	0.92	2.11	13.51	17.52	6.34	39.48
Lebanon	0.04	0.04	0.01	<0.01	0.11	0.26	1.61	2.00	0.73	4.60
Oman	0.03	0.03	0.01	<0.01	0.07	0.15	1.00	1.26	0.42	2.82
Qatar	0.10	0.22	0.06	<0.01	0.39	0.61	5.40	7.56	2.59	16.16
Saudi Arabia	2.60	2.26	0.86	0.09	5.81	15.17	90.88	112.18	40.38	258.61
Syrian Arab Republic	0.21	0.12	0.03	0.01	0.37	1.20	6.11	6.77	2.26	16.34
Turkey	2.22	1.77	0.72	0.18	4.89	12.71	73.13	89.03	32.20	207.07
United Arab Emirates	0.03	0.06	0.02	<0.01	0.12	0.19	1.61	2.27	0.79	4.85
Yemen	0.28	0.15	0.03	0.01	0.47	1.33	5.67	5.82	1.16	13.98

	Incident numbers (in 1000s) 2017 by age groups					Prevalent numbers (in 1000s) 2017 by attained age groups				
Major area, region, country or area	0-14	15-39	40-64	65+	Total	0-14	15-39	40-64	65+	Total
EUROPE	21.39	20.79	13.87	6.31	62.36	126.05	790.94	1,071.27	461.47	2,449.73
Eastern Europe	5.96	6.13	3.78	1.44	17.31	34.84	221.98	300.22	123.85	680.89
Belarus	0.09	0.09	0.06	0.02	0.25	0.53	3.34	4.51	1.88	10.26
Bulgaria	0.09	0.10	0.07	0.03	0.30	0.55	3.53	4.90	2.13	11.12
Czechia	0.36	0.36	0.24	0.11	1.06	2.11	13.42	18.37	8.02	41.91
Hungary	0.28	0.31	0.21	0.09	0.89	1.65	10.87	15.16	6.57	34.25
Poland	1.06	1.24	0.73	0.30	3.33	6.26	42.06	58.15	24.46	130.93
Republic of Moldova	0.05	0.06	0.03	0.01	0.15	0.28	1.88	2.55	0.94	5.64
Romania	0.23	0.24	0.16	0.07	0.70	1.35	8.57	11.75	4.95	26.62
Russian Federation	3.14	3.03	1.84	0.63	8.64	18.31	113.63	151.07	61.03	344.04
Slovakia	0.11	0.13	0.08	0.03	0.35	0.67	4.43	6.09	2.52	13.71
Ukraine	0.54	0.58	0.36	0.14	1.63	3.14	20.26	27.66	11.35	62.41
Northern Europe	5.77	5.18	3.16	1.54	15.65	34.13	208.78	274.44	116.85	634.19
Denmark	0.26	0.24	0.15	0.08	0.72	1.51	9.34	12.48	5.37	28.70
Estonia	0.04	0.03	0.02	0.01	0.10	0.22	1.36	1.82	0.79	4.19
Finland	0.56	0.53	0.33	0.18	1.60	3.36	20.88	27.80	12.21	64.24
Iceland	0.01	0.01	<0.01	<0.01	0.02	0.06	0.36	0.46	0.19	1.06
Ireland	0.28	0.21	0.13	0.05	0.67	1.68	9.67	12.20	4.98	28.54
Latvia	0.02	0.02	0.02	0.01	0.07	0.13	0.84	1.14	0.50	2.62
Lithuania	0.09	0.09	0.06	0.03	0.26	0.50	3.25	4.49	1.95	10.20

Norway	0.32	0.29	0.17	0.07	0.86	1.88	11.68	15.41	6.50	35.47
Sweden	0.75	0.67	0.40	0.21	2.03	4.46	27.26	35.73	15.39	82.83
United Kingdom	3.45	3.09	1.87	0.90	9.30	20.33	124.14	162.92	68.97	376.36
Southern Europe	3.45	3.40	2.67	1.26	10.78	20.41	129.03	180.26	81.23	410.93
Albania	0.04	0.04	0.02	0.01	0.11	0.23	1.46	1.94	0.76	4.38
Andorra	<0.01	<0.01	<0.01	<0.01	0.01	0.01	0.08	0.11	0.05	0.25
Bosnia and Herzegovina	0.04	0.05	0.03	0.01	0.13	0.24	1.61	2.26	0.96	5.07
Croatia	0.11	0.11	0.08	0.04	0.33	0.62	4.03	5.56	2.43	12.64
Greece	0.25	0.26	0.19	0.09	0.79	1.47	9.43	13.16	5.82	29.88
Italy	1.30	1.26	1.07	0.55	4.19	7.70	48.44	68.46	31.85	156.44
Malta	0.01	0.02	0.01	<0.01	0.04	0.08	0.53	0.73	0.31	1.66
Montenegro	0.02	0.02	0.01	<0.01	0.06	0.12	0.78	1.03	0.42	2.35
Portugal	0.19	0.20	0.15	0.07	0.60	1.10	7.14	10.05	4.54	22.83
San Marino	<0.01	<0.01	<0.01	<0.01	<0.01	0.01	0.03	0.05	0.02	0.10
Serbia	0.21	0.21	0.13	0.06	0.60	1.23	7.78	10.44	4.37	23.83
Slovenia	0.05	0.05	0.04	0.02	0.15	0.30	1.92	2.66	1.18	6.07
Spain	1.21	1.16	0.93	0.40	3.69	7.14	44.83	62.50	28.03	142.50
TFYR Macedonia	0.03	0.03	0.02	0.01	0.08	0.15	0.97	1.30	0.50	2.92
Western Europe	6.21	6.07	4.26	2.08	18.62	36.68	231.15	316.35	139.54	723.72
Austria	0.24	0.27	0.18	0.08	0.78	1.43	9.45	13.25	5.83	29.96
Belgium	0.35	0.32	0.21	0.10	0.98	2.09	12.81	17.02	7.30	39.23
France	2.22	1.84	1.19	0.61	5.85	13.11	78.00	101.57	43.59	236.28
Germany	2.61	2.90	2.18	1.07	8.76	15.43	102.04	145.46	65.94	328.87
Luxembourg	0.02	0.02	0.01	<0.01	0.05	0.11	0.69	0.93	0.39	2.12
Monaco	<0.01	<0.01	<0.01	<0.01	<0.01	0.01	0.05	0.06	0.03	0.14
Netherlands	0.59	0.55	0.37	0.17	1.68	3.50	21.64	29.11	12.59	66.84
Switzerland	0.17	0.18	0.12	0.05	0.52	1.00	6.48	8.95	3.87	20.29

	Incident numbers (in 1000s) 2017 by age groups					Prevalent numbers (in 1000s) 2017 by attained age groups				
Major area, region, country or area	0-14	15-39	40-64	65+	Total	0-14	15-39	40-64	65+	Total
LATIN AMERICA AND THE CARIBBEAN	12.16	10.71	4.32	1.11	28.30	68.87	406.01	501.50	178.16	1,154.53
Caribbean	0.12	0.11	0.06	0.02	0.30	0.67	3.87	4.91	1.79	11.25
Antigua and Barbuda	<0.01	<0.01	<0.01	<0.01	<0.01	<0.01	0.03	0.04	0.01	0.08
Bahamas	0.01	0.01	<0.01	<0.01	0.02	0.05	0.30	0.38	0.15	0.87
Barbados	<0.01	<0.01	<0.01	<0.01	0.01	0.02	0.09	0.12	0.05	0.27
Cuba	0.04	0.04	0.03	0.01	0.12	0.25	1.56	2.14	0.90	4.85

Dominica	<0.01	<0.01	<0.01	<0.01	<0.01	0.01	0.03	0.03	0.01	0.08
Dominican Republic	0.02	0.01	<0.01	<0.01	0.03	0.08	0.43	0.50	0.15	1.16
Grenada	<0.01	<0.01	<0.01	<0.01	<0.01	0.01	0.05	0.05	0.02	0.13
Haiti	0.02	0.01	<0.01	<0.01	0.03	0.08	0.32	0.32	0.04	0.75
Jamaica	0.02	0.01	0.01	<0.01	0.04	0.09	0.51	0.63	0.22	1.44
Saint Kitts and Nevis	<0.01	<0.01	<0.01	<0.01	<0.01	<0.01	0.02	0.03	0.01	0.06
Saint Lucia	<0.01	<0.01	<0.01	<0.01	<0.01	0.01	0.06	0.08	0.03	0.17
Saint Vincent and the Grenadines	<0.01	<0.01	<0.01	<0.01	<0.01	0.01	0.04	0.05	0.02	0.12
Trinidad and Tobago	0.01	0.01	0.01	<0.01	0.04	0.08	0.44	0.55	0.18	1.25
Central America	2.94	2.21	0.75	0.17	6.07	16.58	92.56	109.40	37.69	256.23
Belize	0.01	0.01	<0.01	<0.01	0.01	0.04	0.23	0.26	0.09	0.62
Costa Rica	0.01	0.01	0.01	<0.01	0.03	0.08	0.49	0.62	0.23	1.41
El Salvador	0.11	0.08	0.03	0.01	0.23	0.61	3.45	4.07	1.42	9.55
Guatemala	0.37	0.23	0.05	0.01	0.66	1.97	9.97	10.99	3.34	26.26
Honduras	0.18	0.13	0.03	0.01	0.35	1.01	5.51	6.29	2.07	14.88
Mexico	2.14	1.66	0.60	0.14	4.53	12.15	68.98	82.56	29.00	192.70
Nicaragua	0.11	0.08	0.02	0.01	0.23	0.63	3.49	4.08	1.36	9.56
Panama	0.01	0.01	<0.01	<0.01	0.03	0.08	0.45	0.53	0.19	1.25
South America	9.10	8.39	3.52	0.92	21.92	51.61	309.57	387.19	138.68	887.05
Argentina	0.75	0.57	0.23	0.08	1.64	4.32	24.57	29.72	11.03	69.63
Bolivia (Plurinational State of)	0.08	0.05	0.01	<0.01	0.15	0.40	1.99	2.22	0.64	5.26
Brazil	7.42	7.02	2.92	0.73	18.09	41.98	253.66	318.31	113.14	727.09
Chile	0.51	0.47	0.23	0.07	1.28	2.97	18.20	23.36	9.03	53.55
Colombia	0.15	0.13	0.05	0.01	0.35	0.85	4.97	6.14	2.17	14.12
Ecuador	0.06	0.04	0.02	<0.01	0.12	0.35	1.92	2.26	0.79	5.31
Guyana	<0.01	<0.01	<0.01	<0.01	<0.01	<0.01	0.01	0.01	<0.01	0.02
Paraguay	0.02	0.01	<0.01	<0.01	0.04	0.10	0.54	0.63	0.20	1.47
Peru	0.04	0.03	0.01	<0.01	0.09	0.25	1.39	1.65	0.57	3.86
Suriname	<0.01	<0.01	<0.01	<0.01	<0.01	<0.01	<0.01	0.01	<0.01	0.01
Uruguay	0.06	0.05	0.02	0.01	0.15	0.35	2.08	2.61	1.02	6.06
Venezuela (Bolivarian Republic of)	0.01	0.01	<0.01	<0.01	0.02	0.05	0.24	0.29	0.09	0.66

Major area, region, country or area	Incident numbers (in 1000s) 2017 by age groups					Prevalent numbers (in 1000s) 2017 by attained age groups				
	0-14	15-39	40-64	65+	Total	0-14	15-39	40-64	65+	Total
NORTHERN AMERICA	16.36	14.84	8.51	3.44	43.15	95.73	583.52	760.88	309.77	1,749.89
Canada	1.75	1.80	1.12	0.46	5.14	10.32	65.97	89.44	37.65	203.38
United States of America	14.60	13.04	7.39	2.98	38.01	85.41	517.55	671.45	272.12	1,546.52
OCEANIA	1.31	1.17	0.64	0.26	3.39	7.76	47.28	61.21	25.12	141.36
Australia/New Zealand	1.31	1.16	0.64	0.26	3.37	7.72	47.08	60.99	25.05	140.84
Australia	1.10	0.99	0.54	0.22	2.85	6.49	39.79	51.62	21.23	119.14
New Zealand	0.21	0.17	0.10	0.04	0.52	1.22	7.29	9.37	3.82	21.70
Melanesia	0.01	<0.01	<0.01	<0.01	0.01	0.03	0.16	0.18	0.05	0.42
Fiji	<0.01	<0.01	<0.01	<0.01	<0.01	0.01	0.07	0.08	0.02	0.18
Papua New Guinea	<0.01	<0.01	<0.01	<0.01	0.01	0.01	0.06	0.07	0.01	0.16
Solomon Islands	<0.01	<0.01	<0.01	<0.01	<0.01	<0.01	0.01	0.01	<0.01	0.02
Vanuatu	<0.01	<0.01	<0.01	<0.01	<0.01	<0.01	0.02	0.03	0.01	0.06
Micronesia	<0.01	<0.01	<0.01	<0.01	<0.01	<0.01	0.01	0.01	<0.01	0.02
Kiribati	<0.01	<0.01	<0.01	<0.01	<0.01	<0.01	0.01	0.01	<0.01	0.02
Marshall Islands	<0.01	<0.01	<0.01	<0.01	<0.01	<0.01	<0.01	<0.01	<0.01	<0.01
Micronesia (Fed. States of)	<0.01	<0.01	<0.01	<0.01	<0.01	<0.01	<0.01	<0.01	<0.01	<0.01
Nauru	<0.01	<0.01	<0.01	<0.01	<0.01	<0.01	<0.01	<0.01	<0.01	<0.01
Palau	<0.01	<0.01	<0.01	<0.01	<0.01	<0.01	<0.01	<0.01	<0.01	<0.01
Polynesia	<0.01	<0.01	<0.01	<0.01	<0.01	0.01	0.03	0.03	0.01	0.08
Cook Islands	<0.01	<0.01	<0.01	<0.01	<0.01	<0.01	<0.01	<0.01	<0.01	<0.01
Niue	<0.01	<0.01	<0.01	<0.01	<0.01	<0.01	<0.01	<0.01	<0.01	<0.01
Samoa	<0.01	<0.01	<0.01	<0.01	<0.01	<0.01	0.02	0.02	0.01	0.05
Tonga	<0.01	<0.01	<0.01	<0.01	<0.01	<0.01	0.01	0.01	<0.01	0.03
Tuvalu	<0.01	<0.01	<0.01	<0.01	<0.01	<0.01	<0.01	<0.01	<0.01	<0.01

ESM Table 9. Sensitivity analysis: Effects of changing assumptions concerning penalty function, mean duration and assumptions concerning scaling of incidence rates across age groups Deviations from the core model marked in %. For input values see ESM Table 7.

	Core model		Ignoring penalty		Reduced mean duration		Changed incidence scaling 1		Changed incidence scaling 2	
	INC[a]	PREV[a]	INC	PREV	INC	PREV	INC	PREV	INC	PREV
WORLD	234.71	9,004.61	0%	16%	0%	-28%	-20%	-16%	18%	7%
HIC	115.60	4,648.01	0%	4%	0%	-28%	-21%	-17%	22%	9%
Upper MIC	42.21	1,707.07	0%	14%	0%	-28%	-19%	-16%	14%	5%
Lower MIC	69.38	2,434.15	0%	34%	0%	-28%	-18%	-15%	13%	5%
LIC	7.52	215.39	0%	77%	0%	-27%	-15%	-12%	7%	3%
AFRICA	23.13	755.59	0%	52%	0%	-27%	-16%	-13%	9%	4%
ASIA	74.39	2,753.51	0%	26%	0%	-28%	-19%	-15%	13%	5%
EUROPE	62.36	2,449.73	0%	4%	0%	-29%	-22%	-17%	25%	10%
LAC	28.30	1,154.53	0%	13%	0%	-28%	-20%	-16%	14%	5%
NA	43.15	1,749.89	0%	5%	0%	-28%	-21%	-16%	21%	8%
OCEANIA	3.39	141.36	0%	3%	0%	-28%	-21%	-16%	20%	8%

[a] Numbers in 1000s

Abbreviations: INC: Incidence; PREV: Prevalence; HIC: High-income countries; MIC: Middle-income countries; LIC: Low-income countries; LAC: Latin America and the Caribbean; NA: Northern America

ESM Fig. 1. Estimated remaining life-years from onset of type 1 diabetes. Danish data 2015-2017

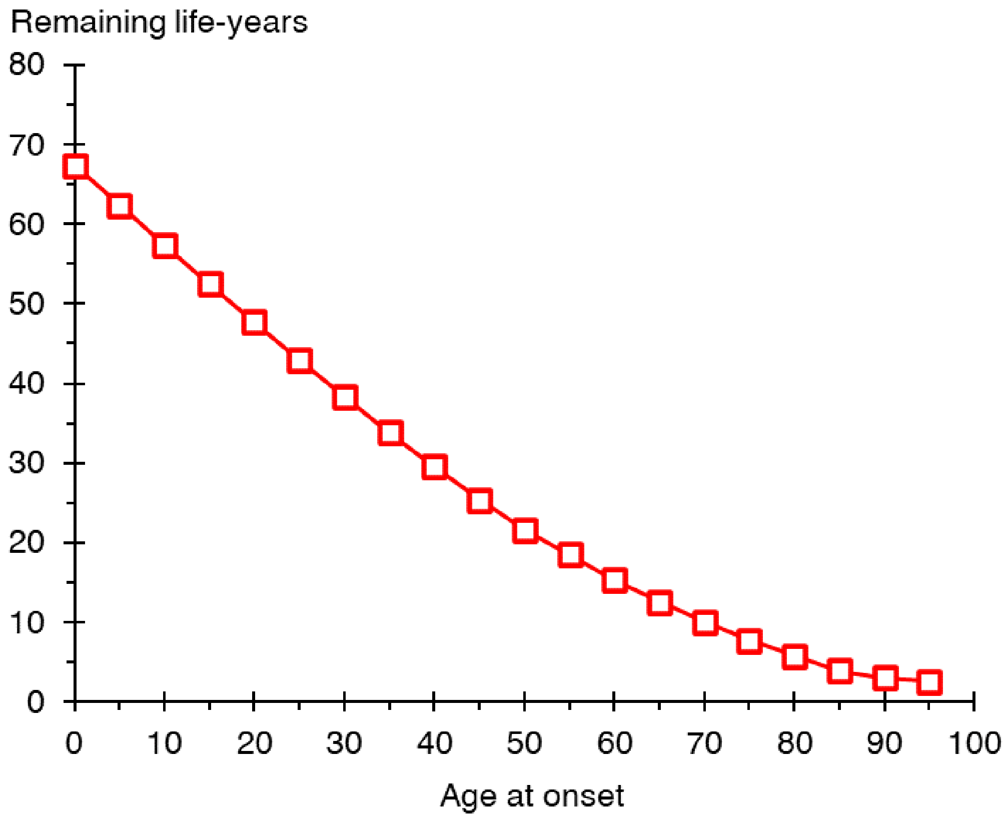

ESM Fig. 2. Illustration of allocating prevalent case numbers to groups by attained age up to age 65 years, see ESM Table 6 for calculations. Example: New Zealand.
A, B, C and D refer to estimated prevalent cases for attained age groups 0-14, 15-39, 40-64 and 65+ years, respectively. The subscripts 0-14, 15-39, 40-64 and 65+ refer to age at onset groups 0-14, 15-39, 40-64 and 65+ years, respectively.

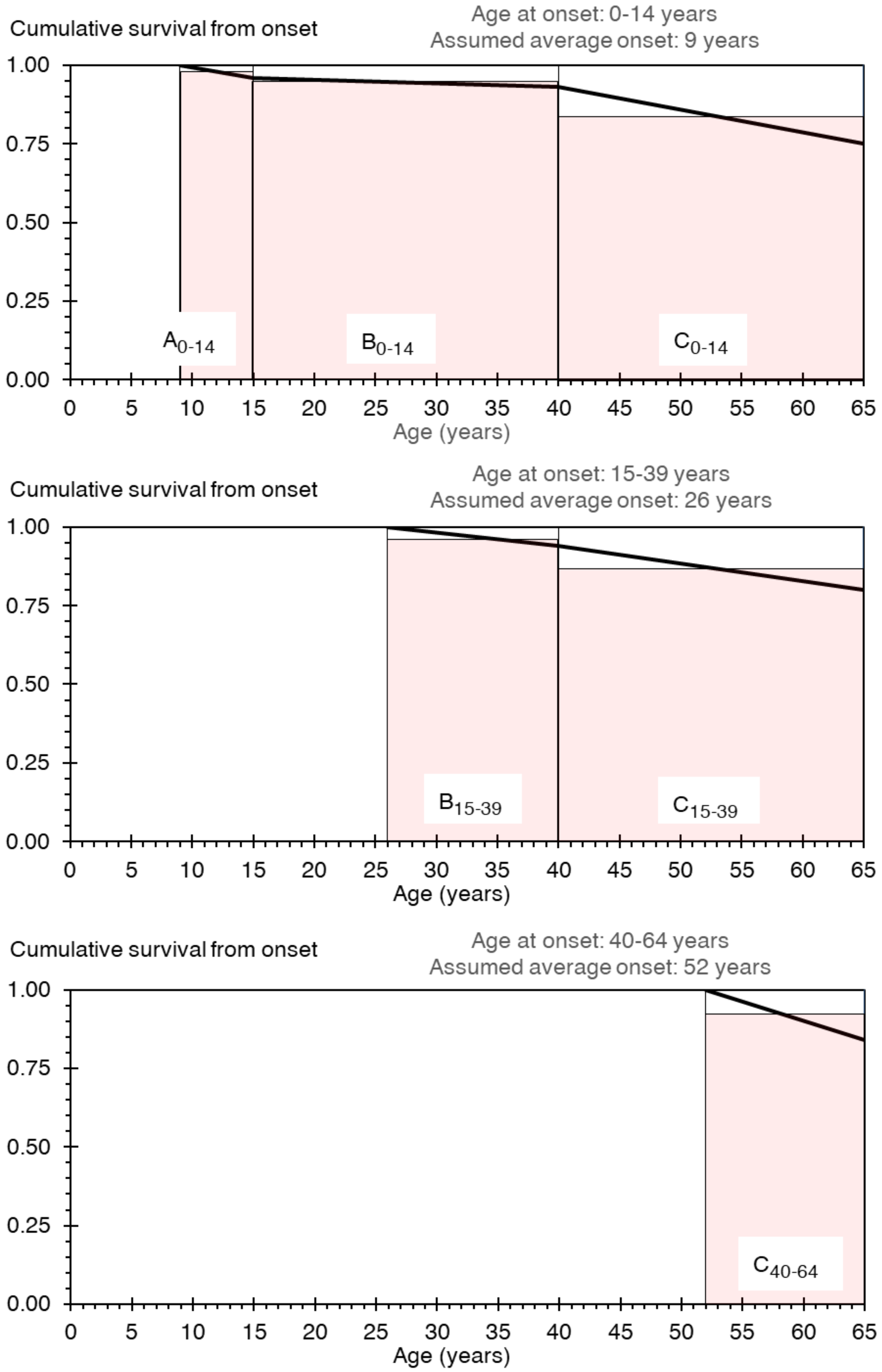

Supplement: Supplementary file 1 — (PDF 706 kb) [file 125_2021_5571_MOESM1_ESM.pdf]
